# Interventions to Support Transitions in Care Among Patients With Cancer: A Scoping Review

**DOI:** 10.1002/cam4.70660

**Published:** 2025-02-28

**Authors:** Negar Rezaei, Jaling Kersen, Abigail Thomas, Stefan Kurbatfinski, Diane Lorenzetti, Khara Marissa Sauro

**Affiliations:** ^1^ Department of Community Health Sciences & O'Brien Institute for Public Health, Cumming School of Medicine University of Calgary Calgary Alberta Canada; ^2^ Department of Surgery, Cumming School of Medicine University of Calgary Calgary Alberta Canada; ^3^ Department of Ocology, Cumming School of Medicine University of Calgary Calgary Alberta Canada

**Keywords:** continuity of care, outcomes, quality of care

## Abstract

**Background:**

The cancer journey from diagnosis through survivorship is complex and involves care from many healthcare providers across a variety of settings. Navigating the transitions between care providers and settings can be improved through interventions. The objective of this study was to map and characterize evidence on interventions to improve transitions in care among patients with cancer.

**Method:**

Six databases were searched to identify relevant studies that described or evaluated interventions to support transitions in care for patients with cancer. Data on the interventions, the type of transition in care, type of cancer, and outcomes (including measure of effectiveness) were abstracted. Data were synthesized and analyzed using descriptive statistics.

**Result:**

Of the 38,876 data sources identified, 150 were included. Most included studies were from the United States and were observational studies exploring interventions to facilitate the transition from treatment to survivorship (followed by interventions for the transition from hospital to home) among patients with breast cancer (followed by gastrointestinal cancers, lung cancers, and hematologic cancers). Interventions that were found to be effective were most commonly those that facilitated the transition from diagnosis to treatment and for the transition from hospital to home.

**Conclusion:**

This comprehensive synthesis is an important resource for those trying to improve transitions in care for patients living with and beyond cancer. Despite the large body of evidence identified, gaps remain; there is a paucity of studies exploring transitions in care during cancer treatment and among some cancers (e.g., brain tumors, head and neck, pancreatic).

## Graphical abstract

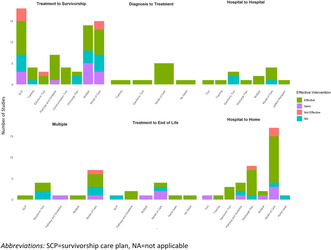

## 1 Introduction

Cancer is the second leading cause of death globally, resulting in 10 million deaths, which is 17.8% of all deaths globally [1]. Cancer also poses a great deal of burden on patients and healthcare systems, resulting in 251.6 million disability‐adjusted life years (DALYs), which is 9.9% of total DALYs, with a 1.5% annual increase [1]. While strategies to reduce the risk of cancer and to effectively treat cancer are important, so too are strategies to reduce the burden of disease among patients with cancer as they move through the cancer care continuum.

The escalating burden of cancer poses challenges for delivering high‐quality healthcare to these patients. The journey of a patient with cancer, from initial diagnosis to survivorship or end‐of‐life care, is complex and dynamic. This complex journey can result in distress among patients with cancer and their families [2, 3, 4] but also places a heavy burden on the healthcare system that provides cancer care [5, 6]. Cancer care involves an intricate network of healthcare providers, including many physicians, nurses, and allied health professionals, among others [7]. The effectiveness of the collaboration between the care team and the integration of their services is critical for delivering high‐quality care. Transitions in care (TiC), the transition of responsibility for patient care between different healthcare providers and healthcare settings, is a particularly challenging period in cancer care [8]. The care journey of patients with cancer requires many TiC. When done well, TiC ensure that patient care is seamless, holistic, and efficiently managed across various stages of living with and beyond cancer [7, 9]. One essential component of effective TiC is the presence of relationships between patients and healthcare providers that are characterized by open communication, mutual trust, and shared decision‐making [9, 10]. Conversely, factors that contribute to poor TiC include inadequate planning among healthcare professionals, lack of patient and family education, limited access to essential services, inappropriate multidisciplinary programs, and a lack of awareness of unmet needs among patients and healthcare providers [9, 10]. These challenges are often exacerbated by constraints such as limited financial resources, insufficient insurance coverage or inadequate access to publicly funded healthcare, and a lack of social support [11, 12]. Poor TiC result in disjointed care, patient dissatisfaction, increased medical errors, and even adverse health outcomes [13].

Interventions to support TiC can improve the continuity of cancer care for patients living with and beyond cancer [8, 14]. Such interventions include the use of integrated electronic health records [15], the establishment of multidisciplinary teams [16], and the introduction of patient navigators [17]. The literature describing and evaluating interventions to improve TiC for patients with cancer is diverse and varied, which can make it challenging for researchers, healthcare providers, and leaders in cancer care delivery to be aware of the effectiveness of interventions to support TiC among patients with cancer. The objective of this scoping review was to map and characterize the literature describing and evaluating interventions to improve TiC among patients with cancer.

## 2 Methods

### 2.1 Protocol and Registration

This scoping review was conducted in accordance with the Joanna Briggs Institute methodology [18] and reported according to the Preferred Reporting Items for Systematic Reviews—Scoping Review Extension (PRISMA‐ScR) [19]. This scoping review did not require ethical approval as it exclusively used published data. This study is part of a larger program of research examining TiC among patients with cancer [20], including a scoping review of TiC in cancer, of which this is a sub‐analysis. The methods for this scoping review have been previously reported in a protocol [8].

### 2.2 Search & Information Sources

The search strategy was developed in collaboration with a medical librarian with expertise in evidence reviews (DL). The search strategy combined subject headings and keywords for two concepts: TiC and the population of interest (patients with cancer) [8]. The search strategy was run in six medical literature databases: MEDLINE, EMBASE, APA PsycINFO, CINAHL, Cochrane CENTRAL, and the Cochrane Database of Systematic Reviews on February 14, 2023. The search was not limited by language, study design, or publication date. The full search strategy is available in Appendix S1.

### 2.3 Eligibility Criteria

All eligible studies from the overarching scoping review were screened for eligibility in this review exploring interventions to improve TiC [20]. In the overarching scoping review, evidence sources were eligible for inclusion if they reported on any TiC among patients with any type of cancer. Additionally, all types of studies including non‐peer‐reviewed articles, such as conference proceedings, viewpoints, editorials, and national or international organizational reports were eligible. Evidence sources were not eligible if they included several diseases or conditions but did not provide stratified data for patients with cancer, only included pediatric patients (evidence sources were eligible if they included both adults and pediatric and stratified data by age group), only examined the transition from pediatric care to adult care, or only reported on changes to TiC related to the COVID‐19 response. For the present study, evidence sources were excluded if they did not describe or evaluate an intervention to improve TiC among patients with cancer.

### 2.4 Selection of Evidence Sources

The selection of evidence sources was managed in Covidence [21], a web‐based software designed for review management. After duplicates were removed, two reviewers independently screened titles and abstracts for eligibility. All reviewers (JK, KS, SK, AT) conducted a calibration exercise to ensure reliable screening; the same 20 studies were independently screened by all reviewers, and agreement was measured using Cohen's kappa; the process was repeated until a Cohen's kappa of 0.8 was achieved. At the title and abstract screening phase, any evidence source deemed eligible by at least one reviewer was moved forward to the full text screening phase. At the full text screening phase, two reviewers independently reviewed all full texts in duplicate for eligibility. Before proceeding with full text screening, the same calibration exercise, as used for title and abstract screening, was conducted. Any conflicts in eligibility between reviewers at the full text screening phase were discussed and resolved. The reason evidence sources were excluded at the full text phase was noted.

### 2.5 Data Charting

A preliminary data extraction spreadsheet was developed by the research team. The data extraction spreadsheet was pilot‐tested by the team, using 20 evidence sources, then revised before beginning data extraction. Two researchers extracted data elements, including bibliographic information for each evidence source (e.g., author, publication date, country, etc.), study design, population characteristics (e.g., age, sex, type of cancer, etc.), information on the TiC, and information on the intervention (e.g., description, outcomes, effectiveness, etc.). One researcher extracted data from each evidence source, which was then audited by a second researcher (JK, SK). Consensus on the extracted data was achieved through discussion. A formal quality assessment of the included evidence sources was not conducted. The data dictionary for the data extraction spreadsheet is provided in Appendix S2.

### 2.6 Data Items

#### 2.6.1 Type of Cancer

The exact description of the type(s) of cancer included in the evidence sources was extracted. For data synthesis, the type of cancer was categorized, and evidence sources that explored multiple types of cancer without specifying the included types of cancer were classified as “multiple” and evidence sources that did not specify the type of cancer were classified as “not specified”

#### 2.6.2 Transitions in Care

The exact description for each TiC was extracted from the evidence sources. For data synthesis, TiC were categorized according to the location from and to; for example, from hospital to home, or from treatment to survivorship. Evidence sources that explored multiple TiC without specifying the included TiC were classified as “multiple” and evidence sources that did not specify the TiC were classified as “not specified.”

#### 2.6.3 Interventions

The exact description of the intervention in the evidence source was extracted. For data synthesis, interventions were categorized into several categories through consensus between two reviewers (NR and KMS). The following categories were developed: model of care (referring to a way of delivering or organizing care which includes protocols, roles, responsibilities, and location/setting), tools (subdivided into electronic referring to any tool intended to be used electronically in any system or digitally, communication tool referring to any tool intended to address issues with communication, and other), training and educational, pathways and guidelines (local, national or international), discharge plan (personalized plan describing the patients' needs prior to discharge from care), patient navigator (a patient who helps other patients navigate cancer care system), survivorship care plan (SCP, a document outlining the care and treatment received and a plan for follow‐up in survivorship), home‐based (care provided in the home), and multiple interventions (Appendix S3).

#### 2.6.4 Outcomes

The outcomes that were used to evaluate the interventions were extracted verbatim from the included evidence sources. For data synthesis, outcomes were categorized as patient‐related (including symptoms, survival and recovery, patient needs, and knowledge), quality of life, satisfaction, system‐related, continuity of care, timeliness, provider‐related, feasibility and accessibility, and qualitative (themes identified in the original articles).

#### 2.6.5 Effectiveness

The effectiveness of interventions was categorized as not effective, semi‐effective, and effective through consensus by two reviewers (NR, KMS). The effectiveness of the interventions was based on changes in the outcomes reported in the included studies; if there was an improvement in all outcomes measured after implementing the intervention, this was considered “effective” whereas if there was an improvement in only some of the outcomes measured, this was considered “semi‐effective” and those with no improvement or worsening of the outcomes measured were considered “not effective” The intention to measure effectiveness (i.e., study design) was also taken into consideration when classifying the effectiveness of the intervention. Evidence sources that did not evaluate the effectiveness of the interventions but rather just described the intervention were categorized as not applicable.

### 2.7 Data Synthesis

The characteristics of the study and patients were synthesized using descriptive statistics (mean with standard deviation (SD), median with interquartile ranges (IQR), frequency with proportions; %). Data was synthesized to describe the types of TiC, types of cancer, types of interventions, outcomes, and the effectiveness. The number (%) of publications in each category for the aforementioned variables was synthesized and mapped across variables. Quantitative data were analyzed using Stata (version 14.0, StataCorp LLC) and visualized using R (Version 4.0.2, R Foundation for Statistical Computing).

## 3 Results

### 3.1 Study Selection

The search identified 38,876 titles and abstracts, of which 26,431 full‐text articles were reviewed for eligibility criteria and 150 evidence sources were included that described or evaluated interventions to improve TiC (Appendix S4). Detailed characteristics of the included studies can be found in Appendix S5.

### 3.2 Study Characteristics

A summary of the included study characteristics is presented in Table 1. The 150 studies represent data from 17 countries, with about half (45.3%) from the United States. The sample size varied from 3 patients [22] to 156,939 patients [23]. The age of the included patients ranged from 18 years old to 88 years old. The included studies were published from 1987 to 2023, with a median year of publication of 2019 (IQR: 2016–2021). The number of evidence sources published before the COVID‐19 pandemic was similar to the number published during and after the COVID‐19 pandemic (49.3%, *n* = 74 vs. 50.7%, *n* = 76); however, when considering the number of years in each group, the rate of publications per year was greater during and after the COVID‐19 pandemic (2.3 publication per year before COVID‐19 vs. 25.3 publication per year during and after COVID‐19). More details on the included evidence sources can be found in Table 1 and Appendix S5.

**TABLE 1:** Summary of included studies.

Variable	Level	*N* (%)	References
Country	United States	68 (45.3%)	[16, 17, 18, 19, 21, 22, 23, 24, 25, 26, 27, 28, 29, 30, 31, 32, 33, 34, 35, 36, 37, 38, 39, 40, 41, 42, 43, 44, 45, 46, 47, 48, 49, 50, 51, 52, 53, 54, 55, 56, 57, 58, 59, 60, 61, 62, 63, 64, 65, 66, 67, 68, 69, 70, 71, 72, 73, 74, 75, 76, 77, 78, 79, 80, 81, 82, 83, 84]
	Canada	20 (13.3%)	[85, 86, 87, 88, 89, 90, 91, 92, 93, 94, 95, 96, 97, 98, 99, 100, 101, 102, 103, 104]
	Australia	15 (10%)	[105, 106, 107, 108, 109, 110, 111, 112, 113, 114, 115, 116, 117, 118, 119]
	United Kingdom	11 (7.3%)	[120, 121, 122, 123, 124, 125, 126, 127, 128, 129, 130]
	France	6 (4%)	[131, 132, 133, 134, 135, 136]
	Denmark	5 (3.3%)	[137, 138, 139, 140, 141]
	Netherlands	5 (3.3%)	[142, 143, 144, 145, 146]
	Japan	5 (3.3%)	[147, 148, 149, 150, 151]
	China	4 (2.7%)	[152, 153, 154, 155]
	Iran	3 (2%)	[156, 157, 158]
	Germany	2 (1.3%)	[159, 160]
	Italy	1 (0.7%)	[161]
	Turkey	1 (0.7%)	[162]
	Singapore	1 (0.7%)	[163]
	South Korea	1 (0.7%)	[164]
	Belgium	1 (0.7%)	[165]
	Sweden	1 (0.7%)	[166]
Study design	Observational	53 (35.3%)	[12, 19, 22, 23, 31, 34, 35, 38, 41, 43, 44, 49, 59, 60, 61, 62, 64, 66, 67, 70, 71, 76, 77, 78, 82, 83, 87, 90, 92, 93, 98, 99, 100, 101, 105, 111, 112, 113, 114, 121, 124, 127, 132, 133, 137, 143, 148, 150, 152, 160, 162, 163, 164]
	Before and after design	24 (16%)	[21, 22, 24, 27, 31, 42, 45, 46, 52, 53, 54, 56, 80, 81, 84, 88, 91, 107, 116, 126, 134, 138, 156, 161]
	Randomized clinical trial	24 (16%)	[16, 18, 29, 50, 57, 72, 74, 76, 85, 86, 95, 104, 119, 120, 139, 140, 141, 142, 145, 153, 154, 157, 159, 166]
	Qualitative	19 (12.7%)	[17, 25, 28, 30, 37, 39, 40, 51, 63, 69, 73, 89, 102, 108, 118, 129, 130, 136, 165]
	Cohort	16 (10.7%)	[47, 48, 68, 75, 79, 103, 109, 110, 122, 125, 131, 144, 147, 149, 151, 155]
	Mixed methods	14 (9.3%)	[33, 41, 65, 94, 96, 97, 106, 115, 116, 117, 128, 135, 146, 158]
Publication time period	Pre‐COVID (1987–2019)	74 (49.3%)	[16, 17, 23, 25, 26, 29, 31, 33, 34, 36, 39, 40, 41, 44, 45, 47, 49, 50, 51, 52, 53, 54, 55, 56, 57, 58, 59, 60, 61, 64, 65, 66, 70, 71, 72, 92, 101, 102, 103, 104, 105, 106, 107, 108, 109, 110, 111, 112, 113, 114, 115, 116, 117, 118, 119, 120, 121, 122, 123, 124, 125, 126, 127, 128, 129, 130, 131, 132, 133, 134, 135, 136, 137, 138]
	COVID (2020–2023)	76 (50.7%)	[22, 27, 28, 30, 32, 35, 37, 38, 42, 43, 46, 48, 62, 63, 67, 68, 69, 93, 139, 140, 141, 142, 143, 144, 145, 146, 147, 148, 149, 150, 151, 152, 153, 154, 155, 156, 157, 158, 159, 160, 161, 162, 163, 164, 165, 166, 167, 168, 169, 170, 171, 172, 173, 174, 175, 176, 177, 178, 179, 180, 181, 182, 183, 184, 185, 186, 187, 188, 189, 190, 191, 192, 193]
Cancer type	Breast	48 (18.7%)	[17, 26, 27, 28, 29, 31, 32, 34, 35, 37, 38, 39, 40, 41, 42, 44, 47, 49, 55, 56, 59, 60, 62, 64, 65, 67, 68, 72, 92, 101, 104, 107, 109, 110, 118, 121, 122, 139, 141, 157, 160, 163, 167, 169, 172, 176, 194]
	Gastrointestinal	40 (15.7%)	[15, 25, 29, 34, 36, 37, 38, 39, 42, 44, 45, 46, 49, 60, 67, 72, 103, 104, 107, 117, 118, 121, 139, 144, 145, 151, 152, 155, 157, 163, 167, 169, 172, 174, 176, 180, 183, 185, 188, 194]
	Lung	28 (10.9%)	[29, 39, 43, 44, 49, 57, 60, 72, 107, 108, 111, 117, 133, 139, 152, 163, 166, 167, 169, 175, 178]
	Hematologic	24 (9.4%)	[16, 29, 39, 51, 53, 56, 58, 60, 67, 117, 118, 119, 126, 134, 135, 146, 157, 169, 170, 181, 182, 183, 189, 195]
	Gynecology	19 (7.4%)	[16, 23, 29, 52, 55, 67, 71, 72, 107, 111, 130, 149, 155, 157, 163, 167, 172, 192, 194]
	Genitourinary	19 (7.4%)	[15, 37, 38, 42, 55, 67, 69, 117, 118, 125, 139, 153, 157, 169, 172, 194]
	Head and Neck	13 (5.1%)	[29, 39, 42, 55, 59, 67, 116, 118, 123, 148, 150, 171, 173]
	CNS	9 (3.6%)	[42, 49, 55, 102, 106, 118, 127, 136, 191]
	Solid Tumor	5 (1.9%)	[55, 58, 117, 182, 183]
	Skin	5 (1.9%)	[67, 111, 118, 167, 183]
	Thoracic	2 (0.8%)	[145, 153]
	Sarcoma	2 (0.8%)	[42, 111]
	Not indicated	42 (16.4%)	[14, 22, 24, 30, 33, 48, 50, 54, 61, 66, 70, 93, 105, 113, 114, 115, 120, 124, 128, 129, 131, 132, 137, 140, 142, 143, 147, 152, 154, 156, 159, 162, 164, 166, 168, 184, 186, 187, 190, 193, 196]
TiC	Treatment to survivorship	68 (45.3%)	[15, 16, 17, 25, 26, 27, 28, 29, 30, 31, 32, 33, 34, 35, 36, 37, 38, 39, 40, 41, 42, 43, 44, 45, 46, 47, 48, 49, 50, 51, 52, 53, 54, 55, 56, 57, 58, 59, 60, 61, 62, 63, 64, 65, 66, 67, 68, 69, 70, 71, 72, 92, 93, 103, 106, 107, 109, 110, 116, 119, 125, 139, 155, 160, 161, 177, 178, 192]
	Hospital to home	37 (24.7%)	[22, 104, 108, 113, 117, 118, 124, 126, 127, 128, 135, 138, 140, 141, 145, 147, 148, 149, 150, 151, 152, 153, 154, 159, 162, 163, 169, 170, 171, 179, 180, 181, 182, 186, 188, 189, 195]
	Multiple	14 (9.3%)	[102, 105, 112, 114, 120, 121, 122, 123, 132, 133, 136, 164, 167, 193]
	Hospital to hospital	13 (8.7%)	[23, 101, 115, 130, 131, 137, 143, 158, 168, 173, 187, 191, 194]
	Treatment to end of life	10 (6.7%)	[111, 142, 146, 156, 157, 165, 172, 176, 183, 190]
	Diagnosis to treatment	8 (5.3%)	[129, 134, 144, 165, 174, 175, 184, 185]
Type of intervention	Model of care	52 (34.7%)	[41, 42, 43, 44, 45, 46, 47, 48, 49, 51, 52, 53, 54, 55, 101, 102, 104, 108, 111, 123, 124, 127, 128, 132, 136, 140, 144, 145, 149, 151, 152, 153, 158, 163, 164, 165, 166, 167, 169, 172, 173, 174, 175, 176, 179, 182, 185, 186, 189, 193, 194]
	Multiple	20 (13.3%)	[56, 57, 58, 59, 60, 61, 62, 63, 64, 65, 66, 67, 68, 69, 105, 131, 135, 162, 187, 190]
	SCP	20 (13.3%)	[17, 24, 25, 26, 27, 28, 29, 30, 31, 32, 33, 34, 35, 36, 37, 38, 39, 40, 122, 183]
	Pathway and guideline	14 (9.3%)	[22, 107, 109, 116, 117, 119, 125, 133, 142, 146, 148, 161, 178, 181]
	E‐Tool	14 (9.3%)	[15, 50, 112, 114, 120, 121, 137, 139, 143, 150, 159, 160, 184, 191]
	Discharge planning	12 (8%)	, [92, 130, 138, 141, 154, 155, 170, 171, 180, 188, 192, 195]
	Training/education	7 (4.7%)	[93, 103, 106, 110, 115, 118, 129]
	Communication tool	4 (2.7%)	[16, 70, 71, 72]
	Tool	2 (1.3%)	[126, 168]
	Home base	2 (1.3%)	[113, 157]
	No detail	2 (1.3%)	[134, 156]
	Patient navigator	1 (0.7%)	[23]

The most frequently studied TiC was the transition from treatment to survivorship (*n* = 68, 45.3%), followed by the transition from hospital to home (*n* = 37, 24.7%), and studies that included multiple TiC (*n* = 14, 9.2%). The TiC least commonly studied was transitions from treatment to end‐of‐life (*n* = 10, 6.7%), and from diagnosis to treatment (*n* = 8, 5.3%; Table 1 and Appendix S5). Most commonly, studies included patients with breast cancer (*n* = 48, 18.7%), followed by gastrointestinal (GI) cancers (*n* = 40, 15.7%), and lung cancer (*n* = 28, 10.9%). Fewer studies explored interventions for TiC among patients with sarcomas (*n* = 2, 0.8%), skin cancers (*n* = 5, 1.9%), and tumors of the central nervous system (*n* = 9, 3.6%; Table 1).

Overall, the most described or evaluated intervention was models of care (*n* = 52, 34.9%), followed by SCP (*n* = 20, 13.3%). There were fewer studies describing or evaluating patient navigators (*n* = 1, 0.7%) or home‐based interventions (*n* = 2, 1.3%; Table 1). More than half (60.5%) of the interventions were classified as effective, while 15.1% were semi‐effective, and 6.6% showed no effect, with 17.8% not reporting on effectiveness (Table 1).

### 3.3 Interventions by TiC

**FIGURE 1:**
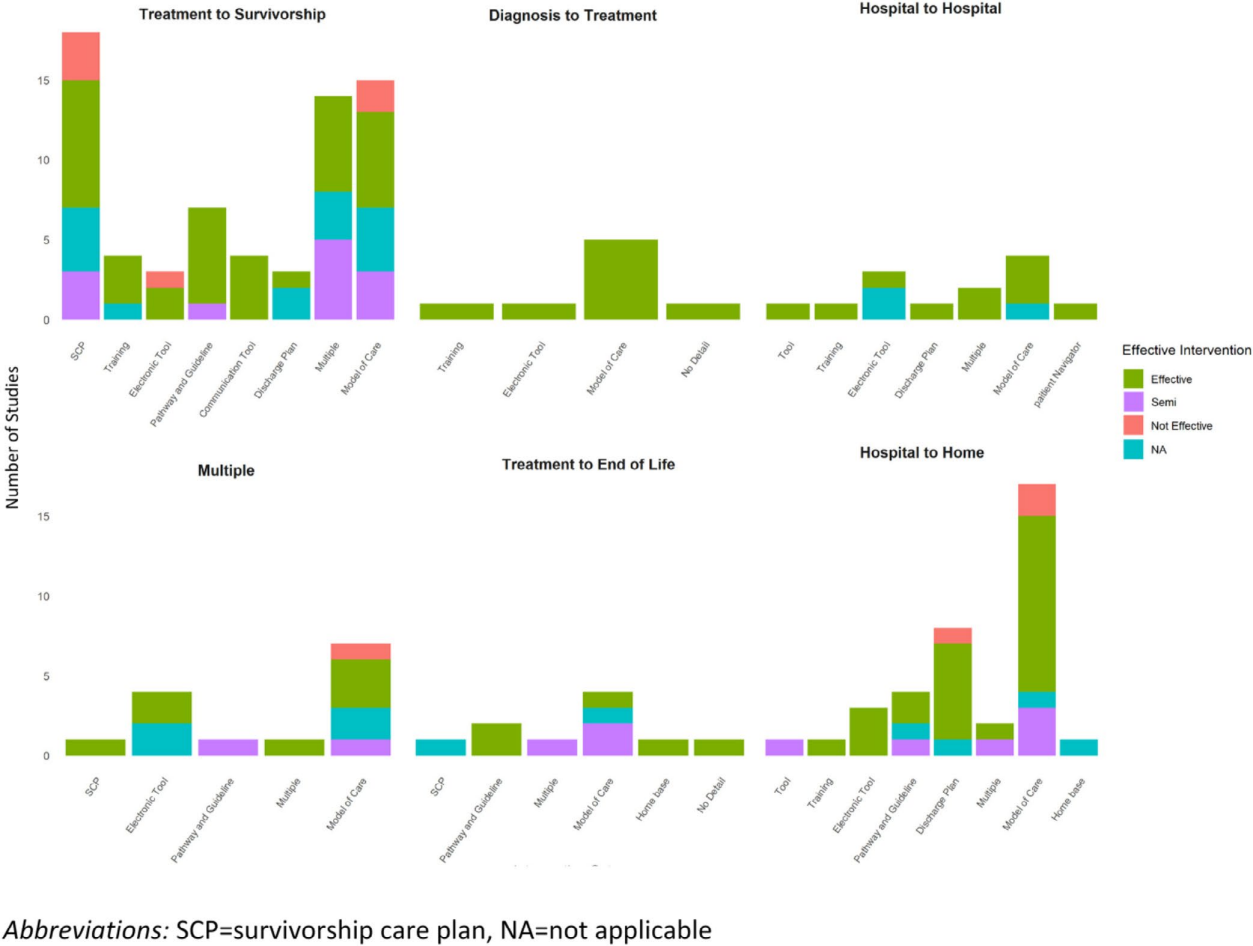
There were more interventions to improve the transition from treatment to survivorship and from the hospital to home, with varied degrees of effectiveness. There were fewer interventions for the transition from diagnosis to treatment, between hospitals, from treatment to the end of life and to improve multiple transitions.

The type of interventions varied by the type of TiC (Figure 1). For the transition from treatment to survivorship, the most common intervention was SCP (*n* = 18, 26.4%) [17, 24, 25, 26, 27, 28, 29, 30, 31, 32, 33, 34, 35, 36, 37, 38, 39, 40], followed by models of care (*n* = 15, 22%) [41, 42, 43, 44, 45, 46, 47, 48, 49, 50, 51, 52, 53, 54, 55], and the use of multiple interventions (*n* = 14, 20.5%) [56, 57, 58, 59, 60, 61, 62, 63, 64, 65, 66, 67, 68, 69]. For all the other transitions, models of care were the most common intervention. The second most common intervention for the transition from treatment to survivorship was models of care; whereas the second most common interventions for the transition from hospital to home were electronic tools, and the second most common intervention for the transition from hospital to home was discharge planning. A summary of the interventions by TiC and type of cancer is presented in Table 2.

**TABLE 2:** Care transition and intervention by cancer type.

Type of cancer (number of articles)	Care transition category	Intervention category (reference)
Breast cancer (*n* = 48)	Hospital to home	Discharge planning [141]
		Model of care [105, 146, 163]
		Training [118]
	Hospital to hospital	Model of care [101, 194]
	Multiple	E‐Tool [121]
	Multiple	Model of care [167]
	Multiple	SCP [122]
	Treatment to end of life	Home base [157]
		Model of care [172, 176]
	Treatment to survivorship	Communication tool [72]
		Discharge planning [92]
		E‐tool [112, 139, 160]
		Model of care [41, 42, 44, 47, 49, 55]
		Multiple [56, 59, 60, 62, 64, 65, 67, 68]
		Pathway and guideline [107, 109]
		SCP [17, 26, 27, 28, 29, 31, 32, 34, 35, 37, 38, 39, 40]
		Training [110]
Gastrointestinal (*n* = 40)	Diagnosis to treatment	Model of care [144, 174, 185]
	Hospital to home	Discharge planning [180, 188]
		E‐Tool [145]
		Model of care [104, 151, 152, 163, 169]
		Pathway and guideline [117]
		Training/education [118]
	Multiple	E‐tool [121]
	Hospital to hospital	Model of care [194]
	Multiple	Model of care [167]
	Treatment to end of life	Home base [157]
		Model of care [172, 176]
		SCP [183]
	Treatment to survivorship	Communication tool [72]
		Discharge planning [155]
		E‐tool [15, 139]
		Model of care [42, 44, 45, 46, 49]
		Multiple [60, 67]
		Pathway and guideline [107]
		SCP [25, 29, 34, 36, 37, 38, 39]
		Training/education [103]
Lung cancer (*n* = 28)	Diagnosis to treatment	Model of care [166, 175, 185]
	Hospital to home	Model of care [119, 152, 163, 169]
		Pathway and guideline [117]
		Training/education [118]
	Multiple	Model of care [167]
	Multiple	Pathway and guideline [133]
	Treatment to end of life	Home base [157]
		Model of care [62, 176]
	Treatment to survivorship	Communication tool [72]
		Discharge planning [155]
		Model of care [42, 43, 44, 49, 55]
		Multiple [57, 60, 63]
		Pathway and guideline [107, 178]
		SCP [29, 39]
Hematologic (*n* = 24)	Diagnosis to treatment	No detail [134]
	Hospital to home	Discharge planning [170, 195]
		Model of care [169, 182, 189]
		Multiple [135]
		Pathway and guideline [117, 181]
		Tool [126]
		Training [118]
	Treatment to end of life	Home base [157]
		Pathway and guideline [146]
		SCP [183]
	Treatment to survivorship	Communication tool [16]
		Model of care [51, 53]
		Multiple [56, 58, 60, 67]
		Pathway and Guideline [119]
		SCP [29, 39]
Gynecology (*n* = 19)	Hospital to home	Model of care [149, 163]
	Hospital to hospital	Discharge planning [130]
		Model of care [194]
		Patient navigator [23]
	Multiple	Model of care [167]
	Treatment to end of life	Home base [157]
		Model of care [172]
	Treatment to survivorship	Model of care [111]
		Communication tool [16, 71, 72]
		Discharge planning [155, 192]
		Model of care [52, 55]
		Multiple [67]
		Pathway and guideline [107]
		SCP [29]
Genitourinary (*n* = 19)	Hospital to home	Model of care [153, 169]
		Pathway and guideline [117]
		Training/education [118]
		Model of care [194]
	Treatment to end of life	Home base [157]
		Model of care [172]
		SCP [183]
	Treatment to survivorship	E‐tool [15, 139]
		Model of care [42, 55]
		Multiple [67, 69]
		Pathway and guideline [125, 161]
		SCP [37, 38, 39]
Head and neck (*n* = 13)	Multiple	Model of care [123]
	Hospital to home	Discharge planning [171]
		E‐tool [150]
		Pathway and guideline [148]
		Training/education [118]
	Hospital to hospital	Model of care [173]
	Treatment to survivorship	Model of care [42, 55]
		Multiple [59, 67]
		Pathway and guideline [116]
		SCP [29, 39]
CNS (*n* = 9)	Hospital to hospital	E‐tool [191]
	Multiple	Model of care [136]
	Hospital to home	Training/education [118]
		Model of care [127]
	Multiple	Model of care [102]
	Treatment to survivorship	Training [106]
		Model of care [42, 55, 65]
Other solid tumor (*n* = 5)	Hospital to home	Model of care [182]
		Pathway and guideline [117]
	Treatment to end of life	SCP [183]
	Treatment to survivorship	Model of care [55]
		Multiple [58]
Skin (*n* = 5)	Hospital to home	Training [118]
	Multiple	Model of care [167]
	Treatment to end of life	Model of care [111]
		SCP [183]
	Treatment to survivorship	Multiple [67]
Thoracic (*n* = 2)	Hospital to home	E‐tool [145]
		Model of care [152]
Sarcoma (*n* = 2)	Treatment to end of life	Model of care [111]
	Treatment to survivorship	Model of care [42]
Not indicated (*n* = 42)	Diagnosis to treatment	E‐tool [184]
		Training/education [129]
	Hospital to home	Discharge planning [138, 154]
	Hospital to home	E‐tool [159]
		Home base [113]
		Model of care [124, 128, 140, 147, 152, 179, 186]
		Multiple [162]
		Pathway and guideline [22]
	Multiple	Model of care [132]
	Hospital to hospital	E‐tool [114, 137, 143]
		Model of care [196]
		Multiple [131, 187]
		Tool [168]
		Training/education [115]
	Treatment to end of life	Model of care [166]
		No detail [156]
		Multiple [190]
		Pathway and guideline [182]
	Treatment to survivorship	Multiple [105]
		Communication tool [70]
		Model of care [48, 50, 54]
		Multiple [61, 66]
		SCP [24, 30, 33]
		Training/education [93]
	Multiple	Model of care [164, 193]
		E‐tool [120]

The effectiveness of each intervention also varied by the type of TiC studied (Figure 1). All of the interventions for the transition from diagnosis to treatment showed improvement in outcomes. The transition between hospitals was the TiC that had the next largest proportion of studies that demonstrated effectiveness (88%) followed by the TiC from hospital to home (69%); whereas studies exploring interventions for multiple TiC and the TiC from treatment to survivorship examined fewer interventions that were found to be effective (50% each).

### 3.4 Interventions by Type of Cancer

**FIGURE 2:**
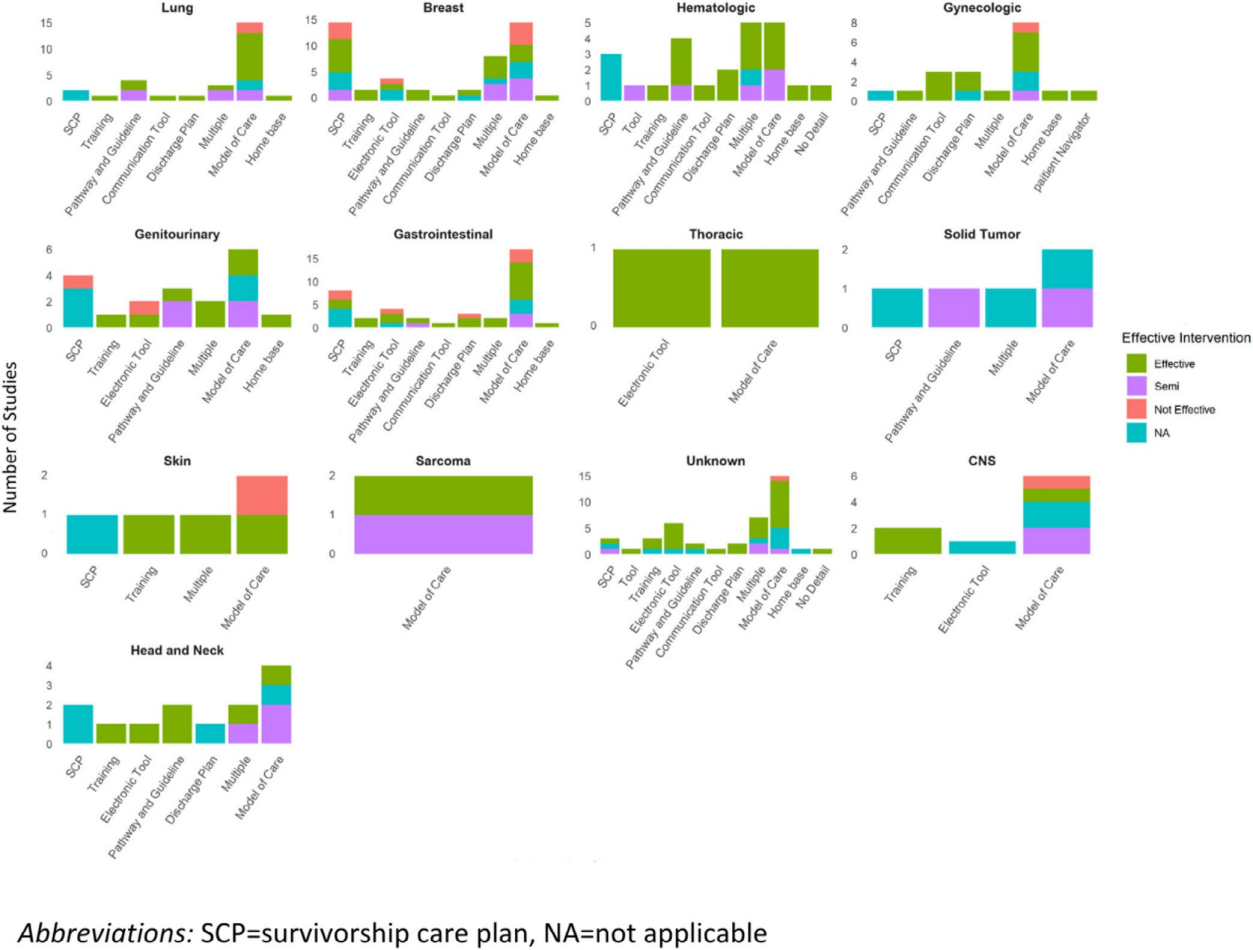
There were more interventions to improve transitions in care among patients with breast and gastrointestinal cancers, with considerable variation in the type of interventions for many types of cancers. The effectiveness of the interventions varied.

The number and type of interventions varied by the type of cancer explored (Figure 2). Studies among patients with hematologic and breast cancers (*n* = 9), and gastrointestinal and lung cancers (*n* = 8, each) had the most variety in the types of interventions, with less variety in interventions examined for sarcomas (*n* = 1), thoracic cancers (*n* = 2), and tumors of the central nervous system (CNS, *n* = 3). With regard to the type of intervention, models of care were evaluated across all cancer types, with tools being the next most explored intervention (*n* = 10, 76.9%), especially electronic tools (*n* = 7, 53.8%). Pathways and guidelines and SCP were also frequently explored (*n* = 9, 69.2% and *n* = 8, 61.5%, respectively). Only studies examining TiC among patients with gynecologic cancers examined patient navigator interventions. More details are provided in Table 2.

The types of interventions that were effective also varied by type of cancer (Figure 2). There was a greater proportion of interventions that were effective for gynecologic cancers (80%) and hematologic cancers (78%) and fewer interventions were found to be effective among studies exploring interventions to improve TiC among patients with multiple types of cancer (54%) and lung cancer (57%). While all interventions were found to be effective for thoracic cancers, there were only two interventions examined. There was also variability between cancers and types of interventions; for example, overall pathways and guidelines were often found to be effective, but fewer studies found pathways and guidelines to be effective in gastrointestinal and genitourinary cancers, compared to head and neck and breast cancer (Figure 2 and Table 2).

### 3.5 Interventions by Outcome

**FIGURE 3:**
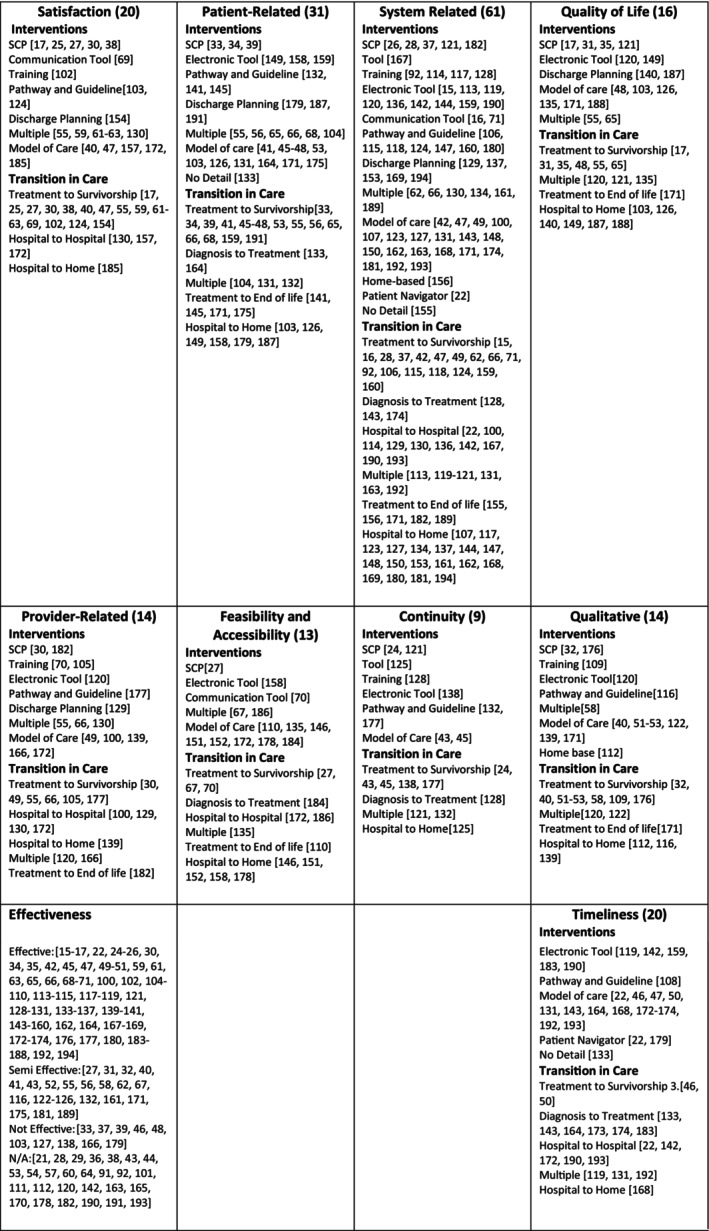
There were many different outcomes used to evaluate the interventions to improve transitions in care. There was variation in the types of outcomes to measure each type of interventions.

The outcomes that were most used to evaluate the interventions were system‐related outcomes (*n* = 61, 28.7%), followed by patient‐related outcomes (*n* = 31, 14.6%), satisfaction and timeliness (*n* = 20, 9.9%; Figure 3). While the outcomes evaluated varied by the TiC of the intervention addressed, interventions for the transition from treatment to survivorship were the most comprehensively evaluated (i.e., using most of the outcome categories), whereas interventions for the transition from treatment to end‐of‐life care were only evaluated using qualitative description, quality of life, feasibility, and patient‐related outcomes (Figure 3). Notably, there were no interventions that were evaluated using patient‐related outcomes for the transition from hospital to hospital, and quality of life was not used as an outcome for interventions for the transition from diagnosis to treatment or from hospital to hospital. Similarly, many of the outcomes (e.g., system related, satisfaction, qualitative) were used consistently to measure all intervention categories (Figure 3), but with an absence of quality‐of‐life measures for training interventions, patient navigation, and communication tools under quality of life.

Among studies that examined the effectiveness of interventions, effectiveness as measured by timeliness (80%) was most commonly found to be effective, followed by feasibility and accessibility (77%). Conversely, studies that examined the effectiveness of interventions using qualitative outcomes were effective in only 29% of cases, and those measured using other measures of effectiveness were only effective in (38%) of studies (Figure 3). More detailed information on each study can be found in Appendix S5.

## 4 Discussion

This scoping review provides a comprehensive resource for researchers, healthcare providers, and decision‐makers interested in improving TiC for patients living with and beyond cancer, by collating 150 evidence sources that describe interventions to facilitate TiC. The included evidence sources describe and evaluate a broad spectrum of interventions designed to improve TiC for patients diagnosed with cancer. The findings of this review highlight the substantial research efforts to date to develop and evaluate interventions to improve TiC for patients with cancer, but also identify some gaps in the evidence. Many of the interventions identified in this review targeted transitions from treatment to survivorship and from hospital to home, suggesting that future research is needed to identify and assess the impact of interventions for other TiC among patients with cancer, such as TiC during cancer treatment. Approximately half of the identified interventions were classified as models of care and SCP, pointing to gaps in the evaluation of other types of interventions. This study also points to the mixed effectiveness of interventions, among many types of cancer and TiC, and on patient‐related outcomes and quality of life. Moreover, the implementation of the interventions was sparsely and variably reported, making it challenging to scale and spread the interventions if found to be effective beyond the context of the individual studies.

The findings of this study suggest that communication and the provision of information play a role in TiC among patients with cancer. Many of the interventions, especially SCP for the transition from treatment to survivorship, focused on the importance of communicating information to both non‐oncology healthcare providers (e.g., family physicians for follow‐up care and surveillance) and patients; notably, many of these interventions were effective [16, 70, 71, 72]. Communication has been acknowledged by others to be a key factor for effective TiC in many settings [73, 74, 75] and is frequently identified as a challenge in delivering high‐quality cancer care by patients and healthcare providers [76, 77, 78, 79, 80]. Effective communication includes gathering and providing information and enabling decisions about treatments and care plans [81, 82]. The absence of any aspect of effective communication can result in patient dissatisfaction (including misunderstandings and ill‐aligned expectations regarding treatment) and can have consequences for clinical outcomes and the safety of care [83, 84, 85]. Having infrastructure to facilitate communication and information exchange is critical for effective TiC [86, 87]. Interventions to support TiC, such as many of those described in the included evidence sources, provide a formal structure that outlines the quantity and quality of information that needs to be communicated. However, evaluating the role of communication and untangling its complex role as a potential modifier in the effectiveness of TiC interventions is warranted.

Evaluating the effectiveness of interventions is critical to adopting them within cancer care. But how do we measure the effectiveness of interventions to improve TiC? The measures used to evaluate the interventions identified in this review were varied, even within a single concept (e.g., outcomes classified as quality of care had varied measurement tools) which makes it challenging to synthesize estimates of effectiveness (i.e., conduct a meta‐analysis). An important first step in measuring the effectiveness of an intervention, including those to improve TiC among patients with cancer, is establishing a common set of quality indicators and measurement standards. Using quality of care models, such as the Donabedian model that includes structure, process, and outcomes [88] and the Institute of Medicine (IOM)'s six domains of quality care (safety, timeliness, effectiveness, efficiency, equitability, patient‐centered) [89], is a good approach to conceptualize the effectiveness of TiC interventions and establish quality indicators. Many of the included studies examined system‐related outcomes such as hospital readmissions and emergency room visits diverted, which align with Donabedian's structure and process domains and the IOM domains of efficiency; fewer focused on clinical outcomes, highlighting an important gap in determining the effectiveness of these interventions. While clinical outcomes were infrequently measured, many studies did focus on patient‐centered measures such as patient satisfaction and quality of life, which are important outcomes for the effectiveness of interventions and may be expanded to include preparedness for TiC, self‐efficacy, and psychosocial outcomes. It is important to note that these patient‐reported outcomes may vary based on the TiC. Given a preponderance of evidence to suggest associations between mismanaged TiC and increases in medical errors or adverse events [74, 83, 90, 91], the sparsity of studies exploring the improvement in the safety of care is particularly notable. More consistent measurement of the effectiveness of interventions to improve TiC among patients with cancer is important and needed to justify implementing the interventions into clinical practice.

While evidence of effectiveness is one of the key facilitators to implementing interventions, the interventions are only as effective as their implementation. The failure to implement should not be conflated with the failure of the intervention. Few of the included evidence sources that implemented the interventions in a real‐world setting (quality improvement or implementation studies) described or evaluated the implementation process, limiting the generalizability beyond a single study's context. Similarly, even fewer evidence sources reported barriers and facilitators (determinants) to implementation; however, of those that did report determinants, the most commonly cited were those related to the knowledge and skills of healthcare providers and lack of resources [33, 39, 65, 68, 92, 93]. These determinants of implementing interventions are common among many clinical contexts (e.g., specialties and settings such as community and in hospitals) [94, 95]. Implementation interventions, such as education and environmental restructuring, which address these determinants, should be considered when developing implementation strategies for effective TiC interventions [96]. Using implementation science approaches to implement and evaluate TiC interventions in care should be considered to improve the adoption of effective interventions to improve TiC among patients with cancer and evade the well‐described gap between what we know is effective and what is being done in the clinical setting [97, 98, 99]. Using implementation science frameworks, theories, and models to outline the determinants of implementing the interventions into clinical practice and evaluating the implementation and the interventions has been found to increase the successful implementation of interventions; therefore, improving care for patients [100]. Similar to using standardized quality indicators to evaluate the effectiveness of interventions, using established implementation science theories, frameworks, and models can help with the scale and spread of the interventions from one setting to others if the interventions are found to be effective. Another important dimension of implementing effective interventions to improve TiC among patients with cancer is the adaptability of the interventions to the needs of patients, especially those belonging to equity‐deserving groups. Ensuring interventions are culturally appropriate and safe for patients of varied racial and ethnic backgrounds, which was not explored in the identified evidence sources.

While this study has several strengths, such as the broad and inclusive eligibility criteria, it has some limitations that should be considered. While efforts were taken to comprehensively search many data sources, it is possible that some studies exploring interventions to improve TiC among patients with cancer were missed. While the broad inclusion of all types of interventions could be considered a strength through the identification of many interventions that may be adopted and adapted by readers. However, it did present challenges in synthesizing the evidence, preventing the ability to perform a quantitative analysis on outcomes and making strong conclusions around the effectiveness of the interventions. Similarly, the evidence synthesis of broad interventions for TiC in cancer care prevented granular details about each intervention and how the interventions can address the complex heterogeneity between different types of cancer, settings, and TiC.

## 5 Conclusion

This comprehensive synthesis of interventions to support transitions in care among patients with cancer provides a resource for those interested in improving transitions in care for patients living with and beyond cancer. Despite the large body of evidence identified, gaps in the evidence remain; there is a paucity of studies exploring transitions in care during cancer treatment and among some cancers (e.g., brain tumors, head and neck, pancreatic).

## Author Contributions

**Negar Rezaei:** formal analysis (lead), visualization (lead), writing – original draft (lead). **Jaling Kersen:** data curation (lead), writing – review and editing (supporting). **Abigail Thomas:** data curation (supporting), writing – review and editing (supporting). **Stefan Kurbatfinski:** data curation (supporting), writing – review and editing (supporting). **Diane Lorenzetti:** conceptualization (supporting), data curation (supporting), methodology (supporting), writing – review and editing (supporting). **Khara Marissa Sauro:** conceptualization (lead), data curation (supporting), formal analysis (supporting), investigation (lead), methodology (lead), project administration (lead), resources (lead), software (supporting), supervision (lead), visualization (supporting), writing – review and editing (lead).

## Ethics Statement

All data presented are publicly available and therefore ethical approval is not required.

## Conflicts of Interest

The authors declare no conflicts of interest.

## Supporting information


Data S1.

Appendix S1.

Appendix S2.

Appendix S3.

Appendix S4.

Appendix S5.

## Data Availability

All data presented in this manuscript are publicly available. The final dataset is available upon reasonable request to the corresponding author.
