# Supplementary material for: Interventions to Support Transitions in Care Among Patients With Cancer: A Scoping Review
Source: Cancer Med. 2025 Feb 28;14(5):e70660. doi: 10.1002/cam4.70660 (PMC11868792; doi:10.1002/cam4.70660)
Supplement: Supplementary file 2 — Appendix S1. [file CAM4-14-e70660-s006.doc]

**Appendix A**. Search Strategy

| **Database** | **Number of references** |
| --- | --- |
| MEDLINE | 8280 |
| Embase | 15287 |
| Cochrane Systematic Review | 96 |
| PsycInfo | 1314 |
| CINAHL | 1061 |
| **TOTAL** | 26038 |
| **With duplicates removed** | 18447 |

Database: Ovid MEDLINE(R) and Epub Ahead of Print, In-Process & Other Non-Indexed Citations and Daily <1946 to February 07, 2020>

Search Strategy:

--------------------------------------------------------------------------------

1 exp Neoplasms/ or (cancer* or carcinoma* or neoplasm* or tumo*).tw. (4121088)

2 Patient Discharg$.tw. (1932)

3 exp Patient Transfer/ (8172)

4 Continuity of Patient Care.tw. (282)

5 continuity of care.tw. (6467)

6 exp Patient Handoff/ (1114)

7 exp Retention in Care/ (105)

8 Aftercare.tw. (2958)

9 (care adj7 transition$).tw. (9350)

10 (quality adj3 transition).tw. (280)

11 treatment delay.tw. (1562)

12 (treatment$ adj1 handoff$).tw. (1)

13 (hospital admission and discharge).tw. (3881)

14 "hospital to home".tw. (2721)

15 "information needs".ti. (1212)

16 "care needs".ti. (2834)

17 "optimi* of rehabilitation".tw. (285)

18 "provision of support".tw. (481)

19 (monitoring adj3 evaluation).tw. (4967)

20 post-treatment.ti. (1853)

21 survivorship.ti. (2704)

22 post treatment surveillance.tw. (231)

23 (discharg$ adj1 plan$).tw. (3806)

24 (discharg$ adj1 process$).tw. (1437)

25 (discharg$ adj1 protocol?).tw. (120)

26 (discharg$ adj1 method$).tw. (350)

27 (discharg$ adj1 transition$).tw. (92)

28 (patient$ adj1 transition$).tw. (1137)

29 (patient$ adj1 discharg$).tw. (12078)

30 (patient$ adj1 transfer$).tw. (4076)

31 (transfer$ adj1 process$).tw. (7842)

32 (transfer$ adj1 pathway$).tw. (2093)

33 (transfer$ adj3 ward$).tw. (468)

34 "transfer$ of patient?".tw. (4143)

35 exp Health Transition/ (1060)

36 (exp animal/ or exp invertebrate/ or animal experiment/ or animal model/ or exp plant/ or exp fungus/) not exp human/ (5303290)

37 or/2-35 (80664)

38 1 and 37 (8370)

39 38 not 36 (8280)

Database: Embase <1974 to 2020 February 07>

Search Strategy:

--------------------------------------------------------------------------------

1 exp Neoplasms/ or (cancer* or carcinoma* or neoplasm* or tumo*).tw. (5180110)

2 patient discharg$.tw. (3592)

3 exp patient transport/ (26226)

4 (continuity adj3 care).tw. (10519)

5 exp clinical handover/ (5942)

6 exp retention in care/ (285)

7 aftercare.tw. (4201)

8 (care adj7 transition$).ti. (4762)

9 (quality adj3 transition).tw. (370)

10 "treatment delay$".tw. (5645)

11 (treatment$ adj2 handoff$).tw. (4)

12 "hospital to home".tw. (3866)

13 "information needs".ti. (1470)

14 "care needs".ti. (3416)

15 "optimi* of rehabilitation".tw. (409)

16 "provision of support".tw. (625)

17 (monitoring adj3 evaluation).tw. (6900)

18 "post treatment".ti. (2704)

19 survivorship.ti. (3908)

20 "post treatment surveillance".tw. (447)

21 (discharg$ adj1 plan$).tw. (5471)

22 (discharg$ adj1 process$).tw. (1769)

23 (discharg$ adj1 protocol?).tw. (242)

24 (discharg$ adj1 method$).tw. (2979)

25 (discharg$ adj1 transition$).tw. (160)

26 (patient$ adj1 transition$).tw. (2451)

27 (patient$ adj1 discharg$).ti. (1440)

28 (patient$ adj1 transfer$).tw. (7216)

29 (transfer$ adj1 process$).tw. (7331)

30 (transfer$ adj1 pathway$).tw. (2093)

31 (transfer$ adj3 ward$).tw. (1080)

32 "transfer$ of patient?".tw. (6779)

33 health transition$.mp. (794)

34 (exp animal/ or exp invertebrate/ or animal experiment/ or animal model/ or exp plant/ or exp fungus/) not exp human/ (5666568)

35 or/2-33 (111372)

36 1 and 35 (15540)

37 36 not 34 (15287)

Database: EBM Reviews - Cochrane Database of Systematic Reviews <2005 to February 4, 2020>

Search Strategy:

--------------------------------------------------------------------------------

1 (Neoplasm or (cancer$ or carcinoma$ or neoplasm$ or tumo$)).mp. [mp=title, abstract, full text, keywords, caption text] (3272)

2 "patient discharg$".mp. [mp=title, abstract, full text, keywords, caption text] (55)

3 "patient transport".mp. [mp=title, abstract, full text, keywords, caption text] (8)

4 (continuity adj3 care).tx. /freq=2 (53)

5 aftercare.tx. /freq=2 (37)

6 (care adj7 transition$).ab. (6)

7 (quality adj3 transition).tx. (1)

8 "treatment delay$".tx. (51)

9 handoff$.mp. (1)

10 handover$.mp. (5)

11 "hospital to home".tx. (106)

12 "information needs".ti. (1)

13 "information need$".ab. (8)

14 "care needs".ti. (1)

15 "care needs".ab. (2)

16 rehabilitation.ab. (217)

17 "provision of support".tx. (16)

18 (monitoring adj3 evaluation).tx. (38)

19 "post treatment".ti. (1)

20 survivorship.ti. (1)

21 survivorship.ab. (5)

22 "post treatment surveillance".tx. (3)

23 (discharg$ adj1 plan$).tx. (43)

24 (discharg$ adj1 process$).tx. (8)

25 (discharg$ adj1 protocol?).tx. (5)

26 discharg$.ab. (224)

27 (discharg$ adj1 transition$).tx. (3)

28 (patient$ adj1 transition$).tx. (5)

29 (patient$ adj1 discharg$).ti. (1)

30 (patient$ adj1 transfer$).tx. (23)

31 (transfer$ adj1 process$).tx. (3)

32 (transfer$ adj3 ward$).tx. (5)

33 "transfer$ of patient?".tx. (19)

34 health transition$.mp. (2)

35 (animal$ or invertebrate or animal experiment or animal model or plant or fungus).mp. [mp=title, abstract, full text, keywords, caption text] (4846)

36 or/2-34 (746)

37 1 and 36 (191)

38 37 not 35 (96)

Database: PsycINFO <1806 to February Week 1 2020>

Search Strategy:

--------------------------------------------------------------------------------

1 exp Neoplasms/ or (cancer* or carcinoma* or neoplasm* or tumo*).tw. (78315)

2 patient discharg$.tw. (297)

3 exp Client Transfer/ (248)

4 (continuity adj3 care).tw. (2480)

5 exp Aftercare/ (1078)

6 (care adj7 transition$).ti. (923)

7 (quality adj3 transition).tw. (168)

8 "treatment delay".tw. (263)

9 "hospital to home".tw. (554)

10 "clinic to home".ti. (19)

11 "information needs".ti. (382)

12 "care needs".ti. (965)

13 "optimi* of rehabilitation".tw. (72)

14 "provision of support".tw. (483)

15 (monitoring adj3 evaluation).tw. (1117)

16 "post treatment".ti. (338)

17 survivorship.ti. (547)

18 "post treatment surveillance".tw. (10)

19 (discharg$ adj1 plan$).tw. (1182)

20 (discharg$ adj1 process$).tw. (199)

21 (discharg$ adj1 protocol?).tw. (12)

22 (discharg$ adj1 method$).tw. (223)

23 (discharg$ adj1 transition$).tw. (36)

24 (patient$ adj1 transition$).tw. (242)

25 (patient$ adj1 discharg$).tw. (2024)

26 (patient$ adj1 transfer$).tw. (715)

27 (transfer$ adj1 process$).tw. (746)

28 (transfer$ adj1 pathway$).tw. (23)

29 (transfer$ adj3 ward$).tw. (81)

30 "transfer$ of patient?".tw. (423)

31 "health transition$".mp. (237)

32 (exp animal/ or exp invertebrate/ or animal research/ or animal model/ or exp "plants (Botanical)"/ or fungus.mp.) not (exp human/ or exp Human Females/ or exp Human Males/) (56530)

33 or/2-31 (14492)

34 1 and 33 (1314)

35 34 not 32 (1314)

**Updated search run on June 14 2023**

| Database | Number of Citations | Date |
| --- | --- | --- |
| Ovid MEDLINE | 11605 | June 14 2023 |
| Embase | 22695 | June 14 2023 |
| APA PsychInfo | 1653 | June 14 2023 |
| CINAHL | 1488 | June 22 2023 |
| Cochrane Systematic Reviews | 110 | June 22 2023 |

**Ovid MEDLINE**

Database(s): **Ovid MEDLINE(R) and Epub Ahead of Print, In-Process, In-Data-Review & Other Non-Indexed Citations and Daily**1946 to June 14, 2023
Search Strategy:

| **#** | **Searches** | **Results** |
| --- | --- | --- |
| 1 | exp Neoplasms/ or (cancer* or carcinoma* or neoplasm* or tumo*).tw. | 4929121 |
| 2 | Patient Discharg$.tw. | 2449 |
| 3 | exp Patient Transfer/ | 9635 |
| 4 | Continuity of Patient Care.tw. | 334 |
| 5 | continuity of care.tw. | 8832 |
| 6 | exp Patient Handoff/ | 1577 |
| 7 | exp Retention in Care/ | 317 |
| 8 | Aftercare.tw. | 3634 |
| 9 | (care adj7 transition$).tw. | 13705 |
| 10 | (quality adj3 transition).tw. | 401 |
| 11 | treatment delay.tw. | 2151 |
| 12 | (treatment$ adj1 handoff$).tw. | 1 |
| 13 | (hospital admission and discharge).tw. | 5405 |
| 14 | "hospital to home".tw. | 3590 |
| 15 | "information needs".ti. | 1476 |
| 16 | "care needs".ti. | 3743 |
| 17 | "optimi* of rehabilitation".tw. | 424 |
| 18 | "provision of support".tw. | 648 |
| 19 | (monitoring adj3 evaluation).tw. | 6646 |
| 20 | post-treatment.ti. | 2531 |
| 21 | survivorship.ti. | 3815 |
| 22 | post treatment surveillance.tw. | 331 |
| 23 | (discharg$ adj1 plan$).tw. | 4667 |
| 24 | (discharg$ adj1 process$).tw. | 2359 |
| 25 | (discharg$ adj1 protocol?).tw. | 218 |
| 26 | (discharg$ adj1 method$).tw. | 485 |
| 27 | (discharg$ adj1 transition$).tw. | 146 |
| 28 | (patient$ adj1 transition$).tw. | 1663 |
| 29 | (patient$ adj1 discharg$).tw. | 16078 |
| 30 | (patient$ adj1 transfer$).tw. | 5307 |
| 31 | (transfer$ adj1 process$).tw. | 10572 |
| 32 | (transfer$ adj1 pathway$).tw. | 2909 |
| 33 | (transfer$ adj3 ward$).tw. | 622 |
| 34 | "transfer$ of patient?".tw. | 5226 |
| 35 | exp Health Transition/ | 1117 |
| 36 | (exp animal/ or exp invertebrate/ or animal experiment/ or animal model/ or exp plant/ or exp fungus/) not exp human/ | 5557535 |
| 37 | or/2-35 | 107624 |
| 38 | 1 and 37 | 11717 |
| 39 | 38 not 36 | 11605 |

**Embase**

Database(s): **Embase**1974 to 2023 June 14

Search Strategy:

| **#** | **Searches** | **Results** |
| --- | --- | --- |
| 1 | exp Neoplasms/ or (cancer* or carcinoma* or neoplasm* or tumo*).tw. | 6610566 |
| 2 | patient discharg$.tw. | 4783 |
| 3 | exp patient transport/ | 33735 |
| 4 | (continuity adj3 care).tw. | 14827 |
| 5 | exp clinical handover/ | 10398 |
| 6 | exp retention in care/ | 1101 |
| 7 | aftercare.tw. | 5138 |
| 8 | (care adj7 transition$).ti. | 6992 |
| 9 | (quality adj3 transition).tw. | 542 |
| 10 | "treatment delay$".tw. | 8263 |
| 11 | (treatment$ adj2 handoff$).tw. | 7 |
| 12 | "hospital to home".tw. | 5209 |
| 13 | "information needs".ti. | 1818 |
| 14 | "care needs".ti. | 4631 |
| 15 | "optimi* of rehabilitation".tw. | 594 |
| 16 | "provision of support".tw. | 846 |
| 17 | (monitoring adj3 evaluation).tw. | 9258 |
| 18 | "post treatment".ti. | 3712 |
| 19 | survivorship.ti. | 5628 |
| 20 | "post treatment surveillance".tw. | 660 |
| 21 | (discharg$ adj1 plan$).tw. | 7060 |
| 22 | (discharg$ adj1 process$).tw. | 2843 |
| 23 | (discharg$ adj1 protocol?).tw. | 401 |
| 24 | (discharg$ adj1 method$).tw. | 4389 |
| 25 | (discharg$ adj1 transition$).tw. | 244 |
| 26 | (patient$ adj1 transition$).tw. | 3790 |
| 27 | (patient$ adj1 discharg$).ti. | 1963 |
| 28 | (patient$ adj1 transfer$).tw. | 9838 |
| 29 | (transfer$ adj1 process$).tw. | 9939 |
| 30 | (transfer$ adj1 pathway$).tw. | 2953 |
| 31 | (transfer$ adj3 ward$).tw. | 1449 |
| 32 | "transfer$ of patient?".tw. | 9022 |
| 33 | health transition$.mp. | 948 |
| 34 | (exp animal/ or exp invertebrate/ or animal experiment/ or animal model/ or exp plant/ or exp fungus/) not exp human/ | 6468046 |
| 35 | or/2-33 | 153033 |
| 36 | 1 and 35 | 22994 |
| 37 | 36 not 34 | 22695 |

**PsycInfo**

Database(s): **APA PsycInfo**1806 to June Week 1 2023
Search Strategy:

| **#** | **Searches** | **Results** |
| --- | --- | --- |
| 1 | exp Neoplasms/ or (cancer* or carcinoma* or neoplasm* or tumo*).tw. | 92581 |
| 2 | patient discharg$.tw. | 406 |
| 3 | exp Client Transfer/ | 299 |
| 4 | (continuity adj3 care).tw. | 3214 |
| 5 | exp Aftercare/ | 1180 |
| 6 | (care adj7 transition$).ti. | 1323 |
| 7 | (quality adj3 transition).tw. | 218 |
| 8 | "treatment delay".tw. | 300 |
| 9 | "hospital to home".tw. | 718 |
| 10 | "clinic to home".ti. | 23 |
| 11 | "information needs".ti. | 455 |
| 12 | "care needs".ti. | 1216 |
| 13 | "optimi* of rehabilitation".tw. | 103 |
| 14 | "provision of support".tw. | 589 |
| 15 | (monitoring adj3 evaluation).tw. | 1330 |
| 16 | "post treatment".ti. | 412 |
| 17 | survivorship.ti. | 688 |
| 18 | "post treatment surveillance".tw. | 11 |
| 19 | (discharg$ adj1 plan$).tw. | 1429 |
| 20 | (discharg$ adj1 process$).tw. | 259 |
| 21 | (discharg$ adj1 protocol?).tw. | 17 |
| 22 | (discharg$ adj1 method$).tw. | 48 |
| 23 | (discharg$ adj1 transition$).tw. | 53 |
| 24 | (patient$ adj1 transition$).tw. | 321 |
| 25 | (patient$ adj1 discharg$).tw. | 2409 |
| 26 | (patient$ adj1 transfer$).tw. | 804 |
| 27 | (transfer$ adj1 process$).tw. | 853 |
| 28 | (transfer$ adj1 pathway$).tw. | 34 |
| 29 | (transfer$ adj3 ward$).tw. | 94 |
| 30 | "transfer$ of patient?".tw. | 471 |
| 31 | "health transition$".mp. | 275 |
| 32 | (exp animal/ or exp invertebrate/ or animal research/ or animal model/ or exp "plants (Botanical)"/ or fungus.mp.) not (exp human/ or exp Human Females/ or exp Human Males/) | 64373 |
| 33 | or/2-31 | 17504 |
| 34 | 1 and 33 | 1654 |
| 35 | 34 not 32 | 1653 |

**CINHAL**

| 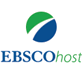 | June 14, 2023 5:11:56 PM |
| --- | --- |

| **#** | **Query** | **Limiters/Expanders** | **Last Run Via** | **Results** |
| --- | --- | --- | --- | --- |
| S42 | S40 AND S41 | Limiters - Exclude MEDLINE records; Human Search modes - Find all my search terms | Interface - EBSCOhost Research Databases Search Screen - Advanced Search Database - CINAHL Plus with Full Text | 1,488 |
| S41 | S1 OR S2 OR S3 | Search modes - Find all my search terms | Interface - EBSCOhost Research Databases Search Screen - Advanced Search Database - CINAHL Plus with Full Text | 774,311 |
| S40 | (TI patient* N2 discharge* OR AB patient* N2 discharge*) OR (S4 OR S5 OR S6 OR S7 OR S8 OR S9 OR S10 OR S11 OR S12 OR S13 OR S14 OR S15 OR S16 OR S17 OR S18 OR S19 OR S20 OR S21 OR S22 OR S23 OR S23 OR S24 OR S25 OR S26 OR S27 OR S28 OR S29 OR S30 OR S31 OR S32 OR S33 OR S34 OR S35 OR S36 OR S37 OR S38 OR S39) | Search modes - Find all my search terms | Interface - EBSCOhost Research Databases Search Screen - Advanced Search Database - CINAHL Plus with Full Text | 70,056 |
| S39 | (MH "health transition+") | Search modes - SmartText Searching | Interface - EBSCOhost Research Databases Search Screen - Advanced Search Database - CINAHL Plus with Full Text | 5,373 |
| S38 | TI transfer* of patient? | Search modes - Find all my search terms | Interface - EBSCOhost Research Databases Search Screen - Advanced Search Database - CINAHL Plus with Full Text | 2,342 |
| S37 | transfer N4 ward | Search modes - Find all my search terms | Interface - EBSCOhost Research Databases Search Screen - Advanced Search Database - CINAHL Plus with Full Text | 261 |
| S36 | transfer* N2 pathway* | Search modes - Find all my search terms | Interface - EBSCOhost Research Databases Search Screen - Advanced Search Database - CINAHL Plus with Full Text | 94 |
| S35 | TI transfer* N2 process* OR AB transfer* N2 process* | Search modes - Find all my search terms | Interface - EBSCOhost Research Databases Search Screen - Advanced Search Database - CINAHL Plus with Full Text | 763 |
| S34 | patient* N2 transfer* | Search modes - Find all my search terms | Interface - EBSCOhost Research Databases Search Screen - Advanced Search Database - CINAHL Plus with Full Text | 6,282 |
| S33 | TI patient* N2 discharge* OR AB patient* N2 discharge* | Search modes - Find all my search terms | Interface - EBSCOhost Research Databases Search Screen - Advanced Search Database - CINAHL Plus with Full Text | 20,646 |
| S32 | patient* N2 transition* | Search modes - Find all my search terms | Interface - EBSCOhost Research Databases Search Screen - Advanced Search Database - CINAHL Plus with Full Text | 3,008 |
| S31 | discharg* N2 transition* | Search modes - Find all my search terms | Interface - EBSCOhost Research Databases Search Screen - Advanced Search Database - CINAHL Plus with Full Text | 389 |
| S30 | discharg* N2 method* | Search modes - Find all my search terms | Interface - EBSCOhost Research Databases Search Screen - Advanced Search Database - CINAHL Plus with Full Text | 2,180 |
| S29 | discharg* N2 protocol? | Search modes - Find all my search terms | Interface - EBSCOhost Research Databases Search Screen - Advanced Search Database - CINAHL Plus with Full Text | 261 |
| S28 | discharg* N2 process | Search modes - Find all my search terms | Interface - EBSCOhost Research Databases Search Screen - Advanced Search Database - CINAHL Plus with Full Text | 1,263 |
| S27 | TI (discharg* N2 plan*) OR AB (discharg* N2 plan*) | Search modes - Find all my search terms | Interface - EBSCOhost Research Databases Search Screen - Advanced Search Database - CINAHL Plus with Full Text | 4,119 |
| S26 | TI "post treatment surveillance" OR AB "post treatment surveillance" | Search modes - Find all my search terms | Interface - EBSCOhost Research Databases Search Screen - Advanced Search Database - CINAHL Plus with Full Text | 95 |
| S25 | TI "survivorship" | Search modes - Find all my search terms | Interface - EBSCOhost Research Databases Search Screen - Advanced Search Database - CINAHL Plus with Full Text | 3,045 |
| S24 | TI "post treatment" | Search modes - Find all my search terms | Interface - EBSCOhost Research Databases Search Screen - Advanced Search Database - CINAHL Plus with Full Text | 799 |
| S23 | monitoring N4 evaluation | Search modes - Find all my search terms | Interface - EBSCOhost Research Databases Search Screen - Advanced Search Database - CINAHL Plus with Full Text | 3,154 |
| S22 | TI "provision of support" OR AB "provision of support" | Search modes - Find all my search terms | Interface - EBSCOhost Research Databases Search Screen - Advanced Search Database - CINAHL Plus with Full Text | 513 |
| S21 | TI "optimi* of rehabilitation" OR AB "optimi* of rehabilitation" | Search modes - Find all my search terms | Interface - EBSCOhost Research Databases Search Screen - Advanced Search Database - CINAHL Plus with Full Text | 105 |
| S20 | TI "care needs" | Search modes - Find all my search terms | Interface - EBSCOhost Research Databases Search Screen - Advanced Search Database - CINAHL Plus with Full Text | 3,318 |
| S19 | TI "information needs" | Search modes - Find all my search terms | Interface - EBSCOhost Research Databases Search Screen - Advanced Search Database - CINAHL Plus with Full Text | 1,173 |
| S18 | TI "hospital to home" OR AB "hospital to home" | Search modes - Find all my search terms | Interface - EBSCOhost Research Databases Search Screen - Advanced Search Database - CINAHL Plus with Full Text | 1,072 |
| S17 | TI "medical appointments and schedules" OR AB "medical appointments and schedules" | Search modes - SmartText Searching | Interface - EBSCOhost Research Databases Search Screen - Advanced Search Database - CINAHL Plus with Full Text | 10,665 |
| S16 | TI "hospital admission and discharge*" OR AB TI "hospital admission and discharge*" | Search modes - SmartText Searching | Interface - EBSCOhost Research Databases Search Screen - Advanced Search Database - CINAHL Plus with Full Text | 414 |
| S15 | treatment* N2 handoff* | Search modes - Find all my search terms | Interface - EBSCOhost Research Databases Search Screen - Advanced Search Database - CINAHL Plus with Full Text | 9 |
| S14 | TI "treatment delay*" OR AB "treatment delay*" | Search modes - Find all my search terms | Interface - EBSCOhost Research Databases Search Screen - Advanced Search Database - CINAHL Plus with Full Text | 1,451 |
| S13 | TI (quality N4 transition*) OR AB (quality N4 transition*) | Search modes - Find all my search terms | Interface - EBSCOhost Research Databases Search Screen - Advanced Search Database - CINAHL Plus with Full Text | 729 |
| S12 | TI "transition* in care" OR AB "transition* in care" | Search modes - Find all my search terms | Interface - EBSCOhost Research Databases Search Screen - Advanced Search Database - CINAHL Plus with Full Text | 289 |
| S11 | TI "transition* of care" OR AB "transition* of care" | Search modes - Find all my search terms | Interface - EBSCOhost Research Databases Search Screen - Advanced Search Database - CINAHL Plus with Full Text | 2,101 |
| S10 | TI (care N8 transition*) OR AB (care N8 transition*) | Search modes - Find all my search terms | Interface - EBSCOhost Research Databases Search Screen - Advanced Search Database - CINAHL Plus with Full Text | 11,738 |
| S9 | TI "aftercare" OR AB "aftercare" | Search modes - Find all my search terms | Interface - EBSCOhost Research Databases Search Screen - Advanced Search Database - CINAHL Plus with Full Text | 1,494 |
| S8 | MM "retention in care+" | Search modes - Find all my search terms | Interface - EBSCOhost Research Databases Search Screen - Advanced Search Database - CINAHL Plus with Full Text | 37,369 |
| S7 | patient handoff* OR handover* | Search modes - Find all my search terms | Interface - EBSCOhost Research Databases Search Screen - Advanced Search Database - CINAHL Plus with Full Text | 2,730 |
| S6 | TI "continuity of patient care" OR AB "continuity of patient care" OR TI "continuity of care" OR AB "continuity of care" OR TI "continuum of care" OR AB "continuum of care" | Search modes - Find all my search terms | Interface - EBSCOhost Research Databases Search Screen - Advanced Search Database - CINAHL Plus with Full Text | 8,473 |
| S5 | (MM "patient transfer+") | Search modes - Find all my search terms | Interface - EBSCOhost Research Databases Search Screen - Advanced Search Database - CINAHL Plus with Full Text | 14,673 |
| S4 | TI "patient discharge*" OR AB "patient discharge*" | Search modes - Find all my search terms | Interface - EBSCOhost Research Databases Search Screen - Advanced Search Database - CINAHL Plus with Full Text | 1,223 |
| S3 | (MH "neoplasms+") | Search modes - Find all my search terms | Interface - EBSCOhost Research Databases Search Screen - Advanced Search Database - CINAHL Plus with Full Text | 654,655 |
| S2 | cancer+ | Search modes - Find all my search terms | Interface - EBSCOhost Research Databases Search Screen - Advanced Search Database - CINAHL Plus with Full Text | 529,029 |
| S1 | neoplasms or oncology or cancer of carcinoma or tumo* | Search modes - Find all my search terms | Interface - EBSCOhost Research Databases Search Screen - Advanced Search Database - CINAHL Plus with Full Text | 698,307 |

Bottom of Form

COCHRANE LIBRARY

Note: Search was previously conducted in OVID’s EBM Reviews - Cochrane Database of Systematic Reviews <2005 to February 4, 2020> but UCalgary has cancelled subscription to this database.

Date Run: 14/06/2023 13:36:52

Comment: Updated search strategy

ID Search Hits

#1 Neoplasm OR (cancer$ OR carcinoma$ OR neoplasm$ OR tumo$) in Cochrane Reviews, Cochrane Protocols 3149

#2 "patient discharge" in Cochrane Reviews, Cochrane Protocols (Word variations have been searched) 108

#3 "patient transport" in Cochrane Reviews, Cochrane Protocols 10

#4 (continuity NEAR/3 care) in Cochrane Reviews, Cochrane Protocols 136

#5 aftercare in Cochrane Reviews, Cochrane Protocols 89

#6 (care NEAR/7 transition):ab in Cochrane Reviews, Cochrane Protocols (Word variations have been searched) 7

#7 (quality NEAR/3 transition) in Cochrane Reviews, Cochrane Protocols 4

#8 "treatment delay" in Cochrane Reviews, Cochrane Protocols (Word variations have been searched) 69

#9 handoff in Cochrane Reviews, Cochrane Protocols (Word variations have been searched) 4

#10 handover in Cochrane Reviews, Cochrane Protocols (Word variations have been searched) 12

#11 "hospital to home" in Cochrane Reviews, Cochrane Protocols 25

#12 "information needs":ti in Cochrane Reviews, Cochrane Protocols 1

#13 "information needs":ab in Cochrane Reviews, Cochrane Protocols 6

#14 "care needs":ti in Cochrane Reviews, Cochrane Protocols (Word variations have been searched) 1

#15 "care needs":ab in Cochrane Reviews, Cochrane Protocols 9

#16 rehabilitation:ab in Cochrane Reviews, Cochrane Protocols 291

#17 "provision of support" in Cochrane Reviews, Cochrane Protocols 18

#18 (monitoring NEAR/3 evaluation) in Cochrane Reviews, Cochrane Protocols 59

#19 "post treatment":ti in Cochrane Reviews, Cochrane Protocols 1

#20 survivorship:ti in Cochrane Reviews, Cochrane Protocols 1

#21 survivorship:ab in Cochrane Reviews, Cochrane Protocols 7

#22 "post treatment surveillance" in Cochrane Reviews, Cochrane Protocols 2

#23 (discharge NEAR/1 plan) in Cochrane Reviews, Cochrane Protocols (Word variations have been searched) 48

#24 (discharge NEAR/1 process) in Cochrane Reviews, Cochrane Protocols (Word variations have been searched) 10

#25 (discharge NEAR/1 protocol) in Cochrane Reviews, Cochrane Protocols (Word variations have been searched) 11

#26 discharge:ab in Cochrane Reviews, Cochrane Protocols (Word variations have been searched) 448

#27 (discharge NEAR/1 transition) in Cochrane Reviews, Cochrane Protocols (Word variations have been searched) 4

#28 (patient NEAR/1 transition) in Cochrane Reviews, Cochrane Protocols (Word variations have been searched) 7

#29 (patient NEAR/1 discharge):ti in Cochrane Reviews, Cochrane Protocols (Word variations have been searched) 1

#30 (patient NEAR/1 transfer) in Cochrane Reviews, Cochrane Protocols (Word variations have been searched) 33

#31 (transfer NEAR/1 process) in Cochrane Reviews, Cochrane Protocols (Word variations have been searched) 5

#32 (transfer NEAR/3 ward) in Cochrane Reviews, Cochrane Protocols (Word variations have been searched) 10

#33 "transfer of patient" in Cochrane Reviews, Cochrane Protocols (Word variations have been searched) 9

#34 (health transition):ti,ab,kw in Cochrane Reviews, Cochrane Protocols (Word variations have been searched) 55

#35 (animal$ or invertebrate or animal experiment or animal model or plant or fungus) in Cochrane Reviews, Cochrane Protocols 4107

#36 #2 OR #3 OR #4 OR #5 OR #6 OR #7 OR #8 OR #9 OR #10 OR #11 OR #12 OR #13 OR #14 OR #15 OR #16 OR #17 OR #18 OR #19 OR #20 OR #21 OR #22 OR #23 OR #24 OR #25 OR #26 OR #27 OR #28 OR #29 OR #30 OR #31 OR #32 OR #33 OR #34 in Cochrane Reviews, Cochrane Protocols 1349

#37 #1 AND #36 378

#38 #37 NOT #35 with Cochrane Library publication date Between Jan 2005 and Jun 2023, in Cochrane Reviews 110
