# Supplementary material for: Interventions to Support Transitions in Care Among Patients With Cancer: A Scoping Review
Source: Cancer Med. 2025 Feb 28;14(5):e70660. doi: 10.1002/cam4.70660 (PMC11868792; doi:10.1002/cam4.70660)
Supplement: Supplementary file 3 — Appendix S2. [file CAM4-14-e70660-s005.docx]

**Appendix B.** Standardized Data Abstraction Form Headings

| **Categorization** | **Abstracted Data** |
| --- | --- |
| **Evidence Source Characteristics** | Author(s), Published Year, Title, Country, Region, Language, Publication Style, Study Design, Methods, Description of Methods, Length of Data Collection, Years of Data Collection |
| **Publication Style** | Journal Article, Dissertation, Conference Proceeding, Book, Editorial, Perspective, Guidelines, Correspondence |
| **Research Objective Categorization** | 1. Patient, Family, Carer, and Healthcare Provider Perspectives on TiC 2. Examination of Risk Factors, Covariates, Protocols or Frequency of TiC 3. Examination or Description of Care Profiles or Patient Characteristics 4. Analysis of Patient, Family, or Healthcare Provider’s needs during TiC 5. Analysis of Delays (Frequency, Risk Factors, Length, and Effect on Patient Outcomes) 6. Evaluation, Review, or Implementation of an Intervention or Program 7. Description, Review, or Analysis focussed on Continuity of Care |
| **Sample Characteristics** | Population Description, Eligibility Criteria, Healthcare Environment Healthcare Professionals Involved, Included Databases, Sample Size, Percentage Female, Percentage Male, Median Age, Age Range, Cancer Type |
| **Cancer Type Categorization** | Multiple, Breast, Colorectal, Head and Neck, Lymphoma, Lung, Pancreatic, Testicular, Thyroid, Melanoma, Endometrial, Brain, Hematologic, Prostate, and Other |
| **Transitions in Care Categorization** | Multiple, Active Treatment to Survivorship, Hospital to Home, Active Treatment to Palliative, Active Treatment to Follow Up, Oncology to Primary Care, Hospital to Hospital, Provider to Provider, Readmission, Discharge, Other Setting to Hospital, Hospital to Other Setting, End of Life TiC, Active Treatment to Posttreatment, Active Treatment to Primary Care, and Other |
| **Transitions in Care Characteristics** | TiC Investigated, Number of TiC, Reason for TiC, Risk Factors for TiC, Outcomes of TiC, Recommendations to Improve TiC, Qualitative Themes Related to TiC, Exemplar Quotes of Qualitative Themes Related to TiC |
| **Intervention Characteristics** | Intervention (Yes/No), Intervention Description, Intervention Evaluation Measurement, Additional Evaluation Measures, Intervention Evaluation Outcome, Recommendations of Intervention |
| **Patient Outcomes** | Patient Outcomes Evaluated (Yes/No), Description of Patient Outcomes Evaluated, Evaluation Measures of Patient Outcomes, Additional Outcomes |
| **Publication Characteristics** | Publication Journal, Journal Impact Factor, Number of Citations, Author Conflicts of Interest (Yes/No) |
