# Supplementary material for: Interventions to Support Transitions in Care Among Patients With Cancer: A Scoping Review
Source: Cancer Med. 2025 Feb 28;14(5):e70660. doi: 10.1002/cam4.70660 (PMC11868792; doi:10.1002/cam4.70660)
Supplement: Supplementary file 4 — Appendix S3. [file CAM4-14-e70660-s003.docx]

**Appendix C:** The description of Multiple Interventions

| **Intervention “Multiple”** | **Description** | **Reference** |
| --- | --- | --- |
| Discharge Planning/Other | Based on a validated (I-PASS) handoff tool, DE-PASS | [39] |
| Electronic Tool/ Guideline Pathway | Readmission risk algorithm identifier, Transitional Care Clinic, Visit guidelines | [77] |
| SCP / Patient Navigator | A survivorship navigator helps facilitate the transition by communicating with patients and PCP’s and collecting data. | [74] |
| SCP / Model Of Care | Bridge to good living Survivor care plan based on unmet needs assessed by multidisciplinary team | [79] |
| SCP / Education | Education, Contact information for long-term follow-up team | [61] |
| SCP / Guidelines& Pathway | Nurse-led development of SCP, aligned with follow-up guidelines, given to patient and GP. | [101] |
| SCP / Education | Patient education | [83] |
| SCP / Education | Orientation session, booklets with information about symptoms, side effects, and new normal | [99] |
| SCP / Model Of Care | SCP, Education seminars, Supportive care referrals, personalized education and self-management mobile App, Decision aids for physicians | [134] |
| SCP / Model Of Care | Nurse Practitioners lead survivorship care model for patient follow up | [76] |
| SCP / Model Of Care | Nurse practitioner led clinic to act as liaison between oncology and GP after treatment | [92] |
| Discharge Planning / Guidelines & Pathway | Guidelines for discharging patients | [97] |
| SCP / Model Of Care | DOMUS( a systematic SPC fast- track transition with a dyadic psychological intervention) | [139] |
| SCP / Model Of Care | Multidisciplinary survivorship care model (Accessible Cancer Care to Enable Support for Cancer Survivors) | [162] |
| SCP / Electronic Tool | SCP E-messages Online resources | [41] |
| Discharge Planning / Tool | Discharge checklist, Handoff template | [22] |
| SCP / Model Of Care | Multidisciplinary survivorship clinic | [26] |
| SCP / Electronic Tool | Creation and delivery of SCPs through the Electronic Medical Record (EMR)to primary care providers in poor, rural, and minority patients | [34] |
| SCP / Electronic Tool | Personalized, patient-facing website tailored follow up to the patients’ PCP and medical oncologist | [73] |
| SCP / Electronic Tool | Multicomponent survivorship program 12-month telehealth program, Interview | [109] |
