# Supplementary material for: Interventions to Support Transitions in Care Among Patients With Cancer: A Scoping Review
Source: Cancer Med. 2025 Feb 28;14(5):e70660. doi: 10.1002/cam4.70660 (PMC11868792; doi:10.1002/cam4.70660)
Supplement: Supplementary file 5 — Appendix S4. [file CAM4-14-e70660-s002.docx]

Studies from databases/registers **(n = 38876)**

**Identification**

Studies sought for intervention **(n = 801)**

Studies excluded **(n = 23056)**

Studies assessed for eligibility **(n = 3375)**

Studies screened **(n = 26431)**

References removed **(n = 12445)**

Duplicates identified manually (n = 40)

Duplicates identified by Covidence (n = 12405)

Studies excluded **(n = 2574)**

1. Not TiC (n = 2205)
2. Could not locate (n = 100)
3. Not patients with cancer (n = 91)
4. COVID only (n = 63)
5. Duplicate data (n = 42)
6. Pediatric only (n = 32)
7. Transition from pediatric to adult care (n=16)
8. Protocol only (n = 13)
9. Other (n=10)

**Screening**

Studies excluded **(n =651)**

1. Not Intervention (n=638)
2. Review (n=11)
3. Duplicate data(n= 2)

**Included**

Studies included in study **(n = 150)**
