# Supplementary material for: Interventions to Support Transitions in Care Among Patients With Cancer: A Scoping Review
Source: Cancer Med. 2025 Feb 28;14(5):e70660. doi: 10.1002/cam4.70660 (PMC11868792; doi:10.1002/cam4.70660)
Supplement: Supplementary file 6 — Appendix S5. [file CAM4-14-e70660-s004.docx]

**Appendix E:** 150 studies describing interventions on Transition in Care among patients with cancer

| **Author** | **Bibliographic Information** | **Country** | **Patient Population** | **Transition in Care** | **Intervention** | **Description** | **Outcomes** | **Effectiveness** |
| --- | --- | --- | --- | --- | --- | --- | --- | --- |
| Adewuyi-Dalton [129] | Psycho-Oncology 1998 | UK | Breast | Treatment  to Survivor | Model of care | Follow-up care in either a hospital practice or general practice | Qualitative | ~  Potential  Slightly greater satisfaction with GP follow-up |
| Ahle [130] | Oncology Research and Treatment^ 2018 | France | Lymphoma | Diagnosis  to  Treatment | No Detail | A series of interventions  (limited description) | Time from: symptoms to diagnosis  diagnosis to treatment | + |
| Alizadeh [157] | Asian Pacific journal of cancer prevention: APJCP 2021 | Iran | Not Indicated | Hospital to Home | Model of Care | Qualitative interview, Review | Validity and Feasibility Qualitative | NA |
| Anderson [44] | Gynecologic Oncology^ 2016 | USA | Cervical | Hospital to Hospital | Patient Navigator | Educational videos and a navigator were introduced to screening clinics | Diagnostic accuracy  Time to colposcopy  Time to treatment | + |
| Aubin [103] | Canadian Family Physician 2015 | Canada | Lung | Treatment to Survivorship | Pathway and Guideline | Standardized communication summaries between GP and oncologist, guidelines for follow-up after diagnosis, priority access to GP | Continuity of care  Interprofessional collaboration  Patient distress  HRU | +  Continuity of care (patient)  0  Continuity of care (GP)  Distress or HRU |
| Aubin [94] | Annals of family medicine 2021 | Canada | Lung | Multiple | Pathway and Guideline | Standardized summary faxed to the FP, Routine Follow up by FP, FP report visit to Oncologist | Continuity of Care, Inter professional collaboration | + |
| Axelsen [136] | Ugeskrift for laeger  2015 | Denmark | Not Indicated | Multiple | Model of Care | Change in monetization of cancer care  Change in case manager function | Time to process  Patient safety | +  Perceived improvement |
| Banala [39] | Journal of Clinical Oncology^ 2016 | USA | Not Indicated | Multiple | Model of Care | Based on a validated (I-PASS) handoff tool, DE-PASS predicted for admission or evaluation. | Reach  ICU transfer  Rescue team call  Satisfaction | + |
| Beadle [46] | American Society of Clinical Oncology 2014 | USA | Head and neck | Treatment to Survivorship | Pathway and Guideline | Pathway for follow-up after surgery consistent with NCCN guidelines and institutional practice | Reach  Appointment duplication | + |
| Benthien [140] | BMJ supportive & palliative care 2020 | Denmark | Multiple | Treatment to Survivorship | Model of Care | Palliative care (SPT) with psychological support | Symptoms, Edmonton Symptom Assessment System | short term +, long term 0 |
| Bhattacharya [77] | Journal of Clinical Oncology ^ 2022 | USA | Not Indicated | Hospital to Home | Multiple | Readmission risk algorithm identifier, Transitional Care Clinic visit guidelines | Readmission | ~ readmission risk algorithm identifier may be useful |
| Birken [12] | Journal of Clinical Oncology^ 2021 | USA | Not Indicated | Treatment to Survivorship | SCP | START (Supporting Transitions After Treatment), a theory-driven, stakeholder-engaged intervention | Qualitative | + |
| Blanchard [113] | Journal of Clinical Oncology^ 2020 | Australia | Not Indicated | Multiple | Model of Care | Oncology nurse practitioner (OND) use in an acute care hospital ED | Time to specialty transfer, Number of admissions versus discharges, Patients representations through ED | + |
| Bodurka [27] | Journal of Clinical Oncology^ 2016 | USA | Gynecology | Hospital to Hospital | Discharge Planning | I-PASS pneumonic to improve transfers between providers from ED, gynecology services and hospitalists | Communication errors  Harm  ICU transfers  Satisfaction (provider) | + |
| Boer [143] | ESMO open 2022 | Netherlands | Testicular | Treatment to Survivorship | Pathway and Guideline | Defining the time points in follow-up and visit location for assessment | Remission status, Failed detection, Failed response | + |
| Brant [74] | Oncology Nursing Forum 2016 | USA | Multiple | Treatment to Survivorship | Multiple | A survivorship navigator helps facilitate the transition by communicating with patients and PCP’s and collecting data. | Quality of Life  Symptoms  Patient satisfaction  PCP satisfaction | ~  Patients and PCPs satisfaction is higher |
| Burkart [79] | Cardiopulmonary Physical Therapy Journal 2019 | USA | Lung | Treatment  to  Survivorship | Multiple | Bridge to good living  Survivor care plan based on unmet needs assessed by multidisciplinary team | Symptoms  Unmet needs | ~  No tests of sig – reduced reported |
| Carpenter-Kellett [86] | Journal of Clinical Oncology 2016 | Canada | Colorectal | Treatment  to  Survivorship | SCP | A transition program build on SCP | Continuity of care (survey) | + |
| Chafee [61] | Biology of Blood and Marrow Transplantation^ 2017 | USA | Multiple | Treatment  to  Survivorship | Multiple | Education, contact information for long-term follow-up team | NA  Description of intervention only | NA |
| Chan [154] | BMC palliative care 2021 | China | Multiple | Treatment to End of life | Model of Care | Palliative Care Support comprehensive assessment, medication review, symptom management and health maintenance | Symptoms, Communication/practical issues | short term +, long term 0 |
| Chare Joo [156] | Medical-Surgical Nursing Journal 2020 | Iran | Multiple | Hospital to Home | Discharge Planning | Training Course, text messages | Quality of life, Severity of symptoms | + |
| Chini [160] | Tumori^ 2019 | Italy | Not Indicated | Hospital to Home | Discharge Planning | Nurse-led early discharge planning program | Hospital stays, Re-admission, effectiveness | + |
| Chiu [36] | Journal of general internal medicine^2021 | USA | Multiple | Treatment  to  Survivorship | Model of Care | Survivorship care established in primary care facility | Patient satisfaction, Qualitative | + |
| Collie [101] | Current Oncology 2014 | Canada | Multiple | Treatment  to  Survivorship | Multiple | Nurse-led development of SCP. Aligned with follow-up guidelines. Given to patient and GP. | Qualitative | ~ |
| Cordier [131] | European Journal of Oncology Pharmacy^ 2018 | France | Not Indicated | Diagnosis to Treatment | Training | Implementing a therapeutic patient education program | Implementation factors  Care coordination | + |
| Coulourides [16] | The American journal of managed care 2017 | USA | Not Indicated | Hospital  to  Home | Model of Care | In-home assessment, follow-up calls after hospital discharge in high-risk patients | Hospital readmit | 0 |
| Coutsouvelis [106] | Supportive Care in Cancer  2010 | Australia | Not Indicated | Hospital  to  Hospital | Training | Education about mouth care, chemotherapy regimen, antibiotics | Number of proper treatments given in ICU | + |
| Coyle [65] | Medical Clinics of North America 1987 | USA | Multiple | Treatment  to  Survivorship | Model of Care | Pain service that provides information and resources | Continuity of care | ? |
| Criticos [111] | Asia-Pacific journal of clinical oncology^2022 | Australia | Breast | Treatment  to  Survivorship | E-Tool | Shared care using the web-based tool | Survival, Cancer Recurrence Identification, Waiting time in the triage of urgent referrals | + |
| Culha [161] | Clinical nursing research 2020 | Turkey | Gynecology | Treatment  to  Survivorship | Discharge Planning | Discharge Training sessions, Patient follow-up | Patient needs after discharges | NA |
| Curcio [83] | Clinical Journal of Oncology Nursing  2012 | USA | Multiple | Treatment  to  Survivorship | Multiple | Patient education | Knowledge  Satisfaction | + |
| Dabholkar [108] | Respirology ^2021 | Australia | Lung | Diagnosis to Treatment | Model of Care | Rapid assessment clinic | Time from referral to diagnosis and treatment, Treatment time, Number of visits | + |
| Dallaire [102] | Canadian journal of neuroscience nursing 2016 | Canada | Neurologic | Hospital  to  Home | Model of Care | Nurse-led service to help patients | Distress  Quality of Life | ~ |
| Daly [58] | Journal of Clinical Oncology^ 2022 | USA | Not Indicated | Hospital to Home | E-Tool | Mobile health intervention to monitor and manage remotely | Symptom/ Feasibility and accessibility | + |
| Davis [33] | Lab Invest 2012 | USA | Not Indicated | Multiple | E-Tool | Web based automated tracking system for handoffs | Compliance  Number of cases | + |
| De Vliegher [164] | Prim Health Care 2015 | Belgium | Not Indicated | Hospital to Home | Home base | Investigation of interventions | Qualitative | NA |
| Duffy [72] | American Journal of Hospice & Palliative Medicine 2018 | USA | Multiple | Hospital  to  Home | Tool | Pharmacist-led discharge checklist including medication reconciliation | Satisfaction  Continuity of care | ~ |
| Duineveld  [144] | Trials 2015 | Netherland | Colorectal | Treatment  to  Survivorship | Model of Care | Surgeon-led care vs. GP led care | NA  Protocol | NA |
| Ehab [52] | Annals of surgical oncology 2022 | USA | Liver | Diagnosis to Treatment | Model of Care | Hepato- biliary-specific weekly multidisciplinary tumor board (HB-MTB) program, (diagnostic algorithm, early appointment, Protocols for each tumor type, Specialist visit) | Time to treatment | + |
| Ehresman  [67] | Journal of Neurosurgery: Spine2021 | USA | Spinal Tumors | Hospital to Hospital | E-Tool | Identify independent risk factors for non-routine discharge and prolonged length of stay (LOS) | Non-routine discharge, prolonged length of stay (LOS) | NA |
| Ekstedt [165] | Studies in health technology and informatics 2014 | Sweden | Breast | Multiple | E-Tool | Added email communication between patient and providers | NA | NA |
| Ezendam [49] | Journal of Cancer Survivorship^ 2014 | USA | Multiple | Treatment  to  End of life | Model of Care | Check-in phone calls from oncology team, optional in-person clinic visit, and a bereavement call | Feasibility | + |
| Faria [99] | Journal of Medical Imaging and Radiation Sciences^ 2017 | Canada | Not Indicated | Treatment  to  Survivorship | Multiple | Orientation session, booklets with information about symptoms, side effects, and new normal | NA  Description only | NA |
| Franzoi [134] | JCO oncology practice 2023 | France | Breast | Treatment  to  Survivorship | Multiple | SCP, Education seminars, Supportive care referrals, personalized education and self-management mobile App, Decision aids for physicians | Patient-perceived satisfaction | + |
| Gandhi [89] | Journal of Clinical Oncology^ 2022 | Canada | Not Indicated | Hospital to Hospital | Model of Care | A physician-assistant led, and physician supervised model | Patient satisfaction, | + |
| Goelz [158] | Journal of Clinical Oncology 2011 | Germany | Multiple | Treatment  to  Survivorship | Communication Tool | Simulation training | Communication | + |
| Goldenberg  [87] | Journal of cancer survivorship: research and practice 2020 | Canada | Colorectal | Treatment  to  Survivorship | Model of Care | Provincial colorectal cancer patient-centered transitions program (Went over plan at visit, sent document to GP, Plan included treatment and follow-up plan) | Patients’ perception of improved continuity of care | + |
| Gorgens [76] | Journal of Thoracic Oncology^ 2021 | USA | Lung | Treatment  to  Survivorship | Multiple | Nurse Practitioners lead survivorship care model for patient follow up | Follow-up care, MD availability, Patient satisfaction | ~ Potential Slightly greater satisfaction with NP follow-up |
| Graboyes [32] | JCO oncology practice 2021 | USA | Head and Neck | Hospital to Hospital | Model of Care | (NDURE) Navigation-Based, Multilevel Intervention, improving patient knowledge and communication, timely appointments | Feasibility, dropout and NDURE session completion, navigator case load, time allocation, patients Satisfaction | + |
| Grainger [112] | Palliative & supportive care 2010 | Australia | Not Indicated | Treatment  to  Survivorship | Communication tool | Communication training – interactive workshop | Satisfaction with workshop | + |
| Grant [92] | Journal of Oncology Practice 2015 | Canada | Breast | Treatment  to  Survivorship | Multiple | Nurse practitioner led clinic to act as liaison between oncology and GP after treatment | Patient satisfaction with program | + |
| Grunfeld [119] | General Practice 1996 | UK | Breast | Treatment  to  Survivorship | Model of Care | Follow-up care in either a hospital practice or general practice | Time to diagnosis  Depression  Anxiety | 0 |
| Gupta [97] | Journal of Clinical Oncology^ 2015 | Canada | Breast | Treatment  to  Survivorship | Multiple | Guidelines for discharging patients | NA  Description | NA |
| Halling [139] | BMC palliative care 2020 | Denmark | Not Indicated | Treatment to End of life | Multiple | DOMUS, a systematic SPC fast- track transition with a dyadic psychological intervention | Incremental cost-effectiveness ratio (ICER), Quality-Adjusted Life Years (QALY) | ~ QALY for caregivers was not significant. |
| Hancock [45] | Oncology Issues 2022 | USA | Not Indicated | Treatment  to  Survivorship | Model of Care | Psychosocial Care, pre/post-treatment clinic visits, educational sessions | Reduction in treatment delays, Positive feedback, psychosocial distress screening, Reducing number of visits | + |
| He [152] | International journal of clinical and experimental medicine 2020 | China | Lymphoma | Hospital to Home | Model of Care | Transitional care by nurse trough phone, or internet | Quality of life | + |
| Hebert [93] | Canadian oncology nursing journal 2017 | Canada | Endometrial | Treatment  to  Survivorship | Communication Tool | Individualized communication about treatment and follow up plans | Acceptability | + |
| Heins [145] | BJGP Open 2018 | Netherland | Prostate | Treatment  to  Survivorship | Pathway and Guideline | Based on national guidelines for follow-up care | Satisfaction with care  General health | +  0 |
| Higashibata [148] | Japanese journal of clinical oncology 2022 | Japan | Multiple | Treatment to End of life | Home base | Home-based nursing service during chemotherapy | Transfer to home care | + |
| Hsueh [80] | Clinical Journal of Oncology Nursing 2016 | USA | Not Indicated | Hospital  to  Home | Model of Care | Advanced nurse facilitated transfer home.  Order sets, communication tool, guidelines. | Length of hospital stay  Readmit | +  0 |
| Huenerberg [50] | Journal of Community and Supportive Oncology 2018 | USA | Breast | Treatment  to  Survivorship | SCP | ISCP and Transition Visit with healthcare professional | Quality Improvement  Satisfaction | + |
| Hulbert-Williams [127] | BMC palliative care 2021 | UK | Multiple | Treatment to End of life | Model of Care | Quantitative effectiveness testing, Qualitative interviews | Quality of life, psychological flexibility, Distress, Perceived effectiveness, Qualitative | ~ Perceived effectiveness and quality of life are improved. |
| Huynh [95] | Palliative medicine^ 2022 | Canada | Not Indicated | Treatment to End of life | No Detail | Patient-informed, root cause improving, after-hours access to hospital and home care | Monthly ED visit rate | + |
| Indoe [126] | The British journal of oral & maxillofacial surgery 2021 | UK | Head and neck | Hospital to Home | Discharge Planning | Patient Concerns Inventory - Ward Discharge | NA, Description of intervention only | NA |
| Jongman [124] | British journal of haematology^ 2021 | UK | Hematology | Hospital to Home | Discharge Planning | Development of Safe Discharge Guideline (SDG), Teaching session, Audit | Quality of discharge follow up plans | + |
| Juarez [166] | Psicooncologia 2013 | USA | Breast | Treatment  to  Survivorship | Training | Bilingual program addressing patient’s education needs | Qualitative | + |
| Karius [66] | Journal of infusion nursing: the official publication of the Infusion Nurses Society 2021 | USA | Not Indicated | Hospital to Home | Pathway and Guideline | Clinical Nurse Specialist Extravasation guidelines for patient management and follow up | NA, Description of intervention only | NA |
| Ke [162] | Journal of Clinical Oncology 2020 | Singapore | Not Indicated | Hospital to Hospital | Multiple | Multidisciplinary survivorship care model (Accessible Cancer Care to Enable Support for Cancer Survivors) | Feasibility, Delivery, Acceptance | + |
| Keenan [69] | Journal of Clinical Oncology^ 2019 | USA | Breast | Treatment to  Survivorship | Discharge Planning | Risk-based Discharge Plan | NA | NA |
| Kenney-Lueptow [40] | Journal of oncology navigation & survivorship 2020 | USA | Not Indicated | Hospital to Home | Model of Care | Nurse Navigation Principles, patient education, relationship-based care, and discharge planning | Patient satisfaction  Length of stay | + |
| Kim [163] | International journal of environmental research and public health 2023 | South Korea | Multiple | Treatment to  Survivorship | Discharge Planning | Discharge Plan Model by nurse on health conditions evaluation, social needs screening toolkit and evaluation, current life situation survey | Patient satisfaction | + |
| Koinberg [128] | BMC nursing 2018 | UK | Head and neck | Multiple | Model of Care | Nurse-led health plan development and support | Qualitative | ~  Potential to improve a transition |
| Kucharczuk [24] | Journal of nursing care quality 2021 | USA | Not Indicated | Hospital to Home | Discharge Planning | Discharge Planning Tool | Readmissions Rate | + |
| Kusajima [146] | American Journal of Hospice & Palliative Medicine 2009 | Japan | Multiple | Treatment to  Survivorship | Model of Care | Specialized home palliative care offering comprehensive services | Quality of Life  Symptoms  Family health status | 0 |
| Kvale [28] | Cancer 2016 | USA | Breast | Multiple | SCP | A coaching encounter using motivational interviewing | Care coordination  Quality of Life | + |
| Ladaique [132] | Programme personnalise d'apres cancer (PPAC): vision des patientes atteintes de cancer du sein et de leur medecin traitant 2021 | France | Breast | Treatment to  Survivorship | SCP | SCP binders, a full range of information on post-cancer care to survivor-specific information and referrals, letters from our anti-cancer center physicians to GP | Comprehensive binder delivery assessment | + |
| Lage [19] | Journal of Clinical Oncology^ 2022 | USA | Multiple | Hospital to Home | Model of care | Supportive oncology care, hospital in the home care model, daily remote monitoring, structured communication with the oncology team | Feasibility | + |
| Lage [42] | Journal of Clinical Oncology^ 2022 | USA | Not Indicated | Hospital to Home | Model of care | Video visit with an oncology nurse practitioner (NP) | Feasibility | + |
| Lee [82] | BMC Cancer  2018 | USA | Multiple | Multiple | E-Tool | EHR-driven registry to facilitate patient transitions, care coordination within a complex care team, coaching team work | Care quality  Care coordination  Qualitative | NA |
| Lefebvre [133] | European Research in Telemedicine 2016 | France | Not Indicated | Multiple | E-Tool | Implementation of Trajectories interface | Transfer rates  Length of stay | + |
| Li [151] | Minerva medica 2022 | China | Colorectal | Hospital to Home | Model of Care | Team systematic management model (Formulate management principles according to the Nursing Standards for Stoma, discharge readiness, transitional care, psycho- logical cognition, education materials | Discharge readiness, Discharge guidance | + |
| Liao [153] | BMC nursing 2022 | China | Head and neck | Hospital to Home | E-Tool | Mobile Health-based nursing model | Patient outcomes (Side effects, Cancer-related fatigue assessment, Quality of life) | + |
| Losk [20] | Journal of Clinical Oncology^ 2013 | USA | Breast | Treatment to  Survivorship | Pathway and Guideline | Standardized appointment scheduling | Time from surgery to chemotherapy | + |
| Malam [100] | Cureus 2020 | Canada | Breast | Treatment to  Survivorship | SCP | Survivorship program entailed a one-on-one education session | Patient satisfaction, Staff satisfaction, Feasibility | ~ patient and staff were in favor but is not feasible in large scale |
| Marston [107] | Australian occupational therapy journal 2023 | Australia | Not Indicated | Hospital to Home | Model of Care | CSNAT-I is a five-stage person- centered model led by caregiver to identify priorities and needs | Qualitative Caregiver Perceptions | + |
| Mateshaytis  [88] | Journal of minimally invasive gynecology 2022 | Canada | Gynecology | Hospital to Home | Model of Care | Four phases of the intervention (Establishing a consistent discharge plan, timely removal of the fully, Patient interview and educational session) | Rate of Same-day Discharge | + |
| Mattessich  [17] | Clinical journal of oncology nursing 2020 | USA | Colorectal | Hospital to Home | Discharge Planning | A goals-to-discharge checklist regarding discharge criteria following colorectal surgery | Recovery | 0 |
| Mayer [71] | Journal of Clinical Oncology 2015 | USA | Multiple | Treatment to  Survivorship | SCP | Comparison of SCP to SCP combined with PCP visit | Confidence in knowledge | NA |
| McBride [53] | American journal of health-system pharmacy 2018 | USA | Multiple | Treatment to  Survivorship | Pathway and Guideline | Using chemotherapy orders for the transition | Reduced cost  Inpatient bed days reduced | + |
| Metcalfe [123] | Laryngoscope investigative otolaryngology 2022 | UK | Head and neck | Hospital to Home | Pathway and Guideline | Novel telescopic pathway, nurse-led clinic for naso-endoscopic examination, consultant-led remote assessment | Cancer conversion rates, Re-referral, Review capacity for endoscopic examination | + |
| Missel [137] | Rehabilitation nursing : the official journal of the Association of Rehabilitation Nurses 2015 | Denmark | Lung | Hospital  to  Home | Model of care | Nurse-led rehabilitation program | Referral to resources | + |
| Montero [38] | Journal of Oncology Practice 2016 | USA | Multiple | Hospital to  Home | Training | Provider education.  Communication and scheduling follow-up | Readmission rates  Cost | + |
| Mooney [25] | Journal of Clinical Oncology^ 2022 | USA | Multiple | Hospital to Home | Model of Care | Rural Community engagement approach (on-ground and telehealth nurse practitioner visits, on-ground registered nurse and physical therapy visits, Cardiovascular remote monitoring) | Feasibility | + |
| Mooney [21] | Journal of Clinical Oncology 2021 | USA | Not Indicated | Hospital to Home | Model of Care | Huntsman at Home program( telehealth nurse practitioner visits, on-ground registered nurse and physical therapy visits, Cardiovascular remote monitoring) | Number of unplanned hospitalizations, costs, length of hospital stays, ICU admissions, and ED visits | + |
| Morisaku [147] | In Vivo 2022 | Japan | Lymphoma | Treatment to End of life | Pathway and Guideline | Early Palliative Care (consulting) | Overall Survival | + |
| Morita [149] | Supportive Care in Cancer 2008 | Japan | Multiple | Treatment to  Survivorship | Pathway and Guideline | Screening tools,  Specialized and on demand palliative care. | Referrals  Identified problems | + |
| Moura [98] | Canadian oncology nursing journal, Revue canadienne de nursing oncologique 2022 | Canada | Not Indicated | Treatment to  Survivorship | SCP | After Cancer Treatment Transition (ACTT) Program | NA, Description of intervention only | NA |
| Nahm [41] | Oncology nursing forum  2019 | USA | Not Indicated | Treatment to  Survivorship | Multiple | SCP  E-messages  Online resources | Symptom burden  Quality of Life | + |
| Nordly [138] | Palliative Medicine 2018 | Denmark | Multiple | Treatment to  Survivorship | Model of Care | Palliative care with psychological support | NA | NA |
| Noteboom  [142] | Journal of cancer survivorship: research and practice 2020 | Netherlands | Multiple | Diagnosis to Treatment | Model of Care | A time out consultation in primary care (TOC) for Shared decision-making (SDM) | Uptake of the TOC | + |
| O’Hea [54] | Journal of oncology navigation & survivorship 2016 | USA | Breast | Treatment to  Survivorship | SCP | Informed SCP plans | Patient and provider  Satisfaction | + |
| O'Hea [75] | Journal of psychosocial oncology 2022 | USA | Breast | Treatment to  Survivorship | SCP | POST Survivorship care planning | Quality of life, Confidence to transition to survivorship | ~ only positive finding is CSI scores were higher for participants who received the POST intervention at 1 month follow up. |
| Ortega [23] | Clinical journal of oncology nursing 2021 | USA | Not Indicated | Hospital to Hospital | Tool | Workflow redesign, hand off tool to improve communication and documentation when transferring patients from the IC to the ED. | Amount of test duplication | + |
| Padgett [81] | Psycho-Oncology ^ 2013 | USA | Not Indicated | Treatment to  Survivorship | SCP | Navigation program  Care plan | Qualitative | ~  Identified barriers and facilitators |
| Page [48] | Journal of Clinical Oncology^ 2014 | USA | Brain | Treatment to  Survivorship | Training | Informing and educating caregivers | Identified caregivers.  Information provided | + |
| Pandya [29] | Journal of Oncology Practice  2019 | USA | Not Indicated | Hospital to Hospital | E-Tool | EMR-based handoff tool. | Compliance  Medication errors  Discharge wait | + |
| Patel [30] | American Journal of Health-System Pharmacy 2017 | USA | Not Indicated | Treatment to  Survivorship | Model of care | Follow-up telephone call program | Completed calls.  Medication adherence | + |
| Patel [35] | Journal of Clinical Oncology^ 2022 | USA | Multiple | Hospital to Home | E-Tool | Telehealth Initiative | 30-days hospital Readmission Rate | + |
| Perfors [141] | Psycho-oncology 2021 | Netherlands | Multiple | Multiple | Model of Care | Time Out consultation with the general practitioner via phone contact | Perceived Cancer treatment decision-making | 0 |
| Petrovic [85] | Journal of medical Internet research 2023 | Canada | Multiple | Treatment to  Survivorship | E-Tool | Web-Based Asynchronous Tool for Communication Between Primary Care Providers and Cancer Specialists | Continuity of care | 0 |
| Phansuwon  [104] | Asia-Pacific journal of clinical oncology 2019 | Australia | Multiple | Treatment to  Survivorship | SCP | Survivorship care plans, interviewed in-person or by phone regarding SCP following | Patient perception | 0 |
| Philip [110] | Neuro-oncology practice  2019 | Australia | Brain | Multiple | Model of Care | Information  Preparation  Coordination  Emotional | Feasibility  Quality of Life  Needs | + |
| Philip [115] | BMC health services research 2021 | Australia | Not Indicated | Treatment to End of life | Model of Care | A multi-site implementation of early palliative care in routine practice | NA, Description of intervention only | NA |
| Phillips [57] | Healthcare (Amsterdam, Netherlands) 2021 | USA | Lung | Diagnosis to Treatment | Model of Care | A novel care delivery model, Lung Cancer Strategist Program (LCSP) | Timeliness of diagnosis and treatment, Care efficiency, Oncologic outcomes | + |
| Poortmans  [135] | Radiotherapy and Oncology^ 2020 | France | Not Indicated | Diagnosis to Treatment | E- Tool | Lean electronic management tool integrated to hospital system | Delay in initiation of therapy | + |
| Porritt [125] | Gut^ 2022 | UK | GI | Diagnosis to Treatment | Model of Care | Early intervention of Clinical Nurse Specialist (CNS) and Cancer Navigator (CN) | Delay, Mean time to diagnosis, Timely and effective management | + |
| Prince [22] | Clinical journal of oncology nursing 2019 | USA | Hematologic | Hospital to Home | Multiple | Discharge checklist.  Handoff template | Follow up appointments.  Communication | + |
| Quinza Real [121] | Critical Care^ 2017 | UK | Hematologic | Treatment to  Survivorship | Model of Care |  | Length of stay  Time on ward  Apache II | + |
| Rariy [60] | Journal of Clinical Oncology^ 2021 | USA | Not Indicated | Multiple | Model of Care | A hybrid delivery model combining in-person visits with Telehealth | Patient travel cost | NA |
| Rio [105] | Australian and New Zealand Journal of Obstetrics and Gynaecology 2017 | Australia | Endometrial | Treatment to  Survivorship | Model of Care | GP model of follow-up care during clinical handover | Qualitative  Communication  Referrals | + |
| Roy [68] | Journal of Clinical Oncology^ 2022 | USA | Not Indicated | Hospital to Hospital | E-Tool | A “Consult to Nurse Navigation” order built into the electronic medical record (EMR) for ER providers | Diagnostic process, Timeliness to care | NA |
| Russell [116] | Journal of cancer survivorship: research and practice 2019 | Australia | Multiple | Hospital to Home | Pathway and Guideline | Referral Pathway to community care | Qualitative | ~ |
| Russell [114] | Journal of cancer survivorship: research and practice 2020 | Australia | Multiple | Treatment to End of life | SCP | A Good Life Cancer Survivorship Program (GLCS program) is a community- based model of unmet supportive care needs | Service utilization; Interviews with healthcare professionals | NA |
| Rutkowski  [91] | Canadian oncology nursing journal, Revue canadienne de nursing oncologique 2021 | Canada | Breast | Treatment to  Survivorship | SCP | Comparing personalized with standard survivorship care plans | Perceived knowledge, Patient activation | + (both are effective) |
| Salner [37] | Psycho-Oncology^ 2012 | USA | Breast | Treatment to  Survivorship | SCP | SCP  Treatment summary | Quality of Life  Patient satisfaction | + |
| Sanft [26] | Psycho-Oncology^ 2015 | USA | Not Indicated | Multiple | Multiple | Multidisciplinary survivorship clinic | Patient distress | + |
| Schnabel  [159] | Oncology research and treatment 2022 | Germany | Not Indicated | Treatment to End of life | Pathway and Guideline | Standardized screening for palliative care needs on elective admission | Physical symptoms, Psychosocial distress of cancer patients | + |
| Sebring [78] | Journal of managed care & specialty pharmacy 2020 | USA | Multiple | Hospital to Home | Model of Care | Pharmacist-Led Comprehensive Chemotherapy Consultation Services (3CS) | Outpatient Appointment Adherence | ~ The unadjusted percentage of appointments attended was significant |
| Shimada  [150] | American Journal of Hospice & Palliative Medicine 2017 | Japan | Multiple | Treatment to  Survivorship | Communication Tool | Rounds when admitted and at discharge | Number of visits | + |
| Sisler [90] | J Clinical Oncology ^ 2017 | Canada | Colorectal | Treatment to  Survivorship | SCP | SCP and transitional appointments | Quality of life | + |
| Smith [64] | Journal of cancer survivorship: research and practice 2022 | USA | Multiple | Treatment to  Survivorship | SCP | Survivorship care planning, Over plan visit, | Descriptive report on intervention fidelity and protocol deviations | NA |
| Smith [63] | Academic medicine 2021 | USA | Not Indicated | Treatment to  Survivorship | Training | Medical Education Course for primary care physicians (PCPs) | Course effectiveness | NA |
| Snyder [18] | Journal of the National Cancer Institute 2022 | USA | Multiple | Treatment to  Survivorship | SCP | comparing three plan-recommended care (mailed plan, plan delivered during a 1-time transition visit, plan delivered during a plus 6-month follow-up visit) | 12 months receive of recommended care | 0 (No difference) |
| Soulia [62] | Journal of the Advanced Practitioner in Oncology 2019 | USA | Multiple | Treatment to  Survivorship | SCP | SCP in community-based oncology | Knowledge | + |
| Sussman  [84] | Journal of Clinical Oncology^ 2012 | Canada | Multiple | Hospital  to  Home | Model of Care | Nurse-led supportive care program.  Supportive care assessment, access to telephone support, link to community resources | Unmet needs  Quality of life | 0 |
| Tamri [155] | Journal of Holistic Nursing & Midwifery 2022 | Iran | Breast | Hospital to Home | Discharge Planning | training, counseling, emotional support, and follow-up | Quality of Life | + |
| Tawfik [34] | Journal of Clinical Oncology^ 2020 | USA | Multiple | Treatment to  Survivorship | Multiple | Creation and delivery of SCPs through the Electronic Medical Record (EMR)to primary care providers in poor, rural, and minority patients | Number of patients and PCPs received SCPs | + |
| Taylor [117] | European journal of oncology nursing 2018 | Australia | Lymphoma | Treatment to  Survivorship | Model of Care | Nurse-led survivorship clinic | Qualitative | ~ |
| Taylor [122] | European Journal of Oncology Nursing 2013 | UK | Colorectal | Treatment to  Survivorship | Training | Weekly support group and education sessions | Patient satisfaction | + |
| Tepper [59] | Smith College Studies Social Work 2003 | USA | Neurologic | Multiple | Model of Care | Access to education, psychosocial support throughout care | NA  Description | NA |
| Terrell [47] | Biology of Blood and Marrow Transplantation^ 2020 | USA | Hematologic | Hospital  to  Home | Pathway and Guideline | Orders for follow-up at discharge via EMR | Readmit | + |
| Thompson [120] | European Journal of Oncology Nursing 2014 | UK | Breast | Treatment to  Survivorship | SCP | Four weekly meetings to prepare for discharge. | Anxiety | 0 |
| Tortorella [43] | Journal of healthcare management 2011 | USA | Breast | Hospital  to  Hospital | Model of Care | Change from ED to unit as place for patient to present | Compliance with documentation | + |
| Tremblay  [96] | BMC health services research 2017 | Canada | Not Indicated | Treatment to  Survivorship | Model of Care | Risk based coordinated care model | Qualitative  Patient outcomes | NA |
| Vaswani [70] | The American journal of emergency medicine 2023 | USA | Multiple | Hospital to Hospital | Model of Care | Multidisciplinary Rapid assessment service clinic (RAS), follow up, diagnosis, treatment for ED patients with suspected malignancies | Average time to clinic appointment, Average time to diagnosis, the final diagnosis based on biopsy. | NA |
| Wallner [73] | Breast cancer research and treatment 2021 | United States | Breast | Treatment to  Survivorship | Multiple | Personalized, patient-facing website tailored follow up to the patients’ PCP and medical oncologist | Feasibility, Acceptability | + Acceptability, ~ accessing the provider letters, and EMR integration issues are a potential barrier |
| Warsame [51] | Journal of oncology practice  Journal of oncology practice | USA | Hematologic | Hospital  to  Home | Discharge Planning | Multidisciplinary discharge huddle and checklist. | Appropriate follow-up  Appropriate medication | + |
| White [118] | Asia-Pacific journal of clinical oncology 2021 | Australia | Multiple | Hospital to Home | Model of Care | CNs visiting patients in their home, Chemotherapy Symptom Assessment | Number of Unplanned presentations | + |
| Xu [55] | BMC cancer 2023 | USA | Multiple | Treatment to  Survivorship | E-Tool | Web-based PRISMS contain educational material, Personalized feedback on care, Reporting platform, Management strategy, follow up | Recruitment, Retention rates, Number of ostomy-related ER visits, Readmissions, Referrals, Follow-up visits | + |
| Yates [109] | BMJ open 2022 | Australia | Prostate | Treatment to  Survivorship | Multiple | Multicomponent survivorship program, 12-month telehealth program, Interview | Patient report care experience improvements | + |

^ = conference abstract only; 0 = not effective; ~ = neutral/inconclusive; + = effective, GP=general practitioner/family physician; HRU=healthcare resource utilization; ED=emergency department; SCP=survivorship care plan; GI=gastrointestinal; EMR=electronic medical record, PCP=primary care provider
